# Psychometric Properties and Validation of the EMOTICOM Test Battery in a Healthy Danish Population

**DOI:** 10.3389/fpsyg.2019.02660

**Published:** 2019-12-03

**Authors:** Vibeke H. Dam, Christa K. Thystrup, Peter S. Jensen, Amy R. Bland, Erik L. Mortensen, Rebecca Elliott, Barbara J. Sahakian, Gitte M. Knudsen, Vibe G. Frokjaer, Dea S. Stenbæk

**Affiliations:** ^1^Neurobiology Research Unit, The Neuroscience Centre, Copenhagen University Hospital Rigshospitalet, Copenhagen, Denmark; ^2^Faculty of Health and Medical Sciences, University of Copenhagen, Copenhagen, Denmark; ^3^Neuroscience and Psychiatric Unit, The University of Manchester, Manchester, United Kingdom; ^4^Department of Public Health and Center for Healthy Aging, University of Copenhagen, Copenhagen, Denmark; ^5^Department of Psychiatry, University of Cambridge, Cambridge, United Kingdom; ^6^Behavioural and Clinical Neuroscience Institute, University of Cambridge, Cambridge, United Kingdom

**Keywords:** EMOTICOM, affective cognition, social cognition, hot cognition, psychometrics, neuropsychological test battery

## Abstract

Disruptions in hot cognition, i.e., the processing of emotionally salient information, are prevalent in most neuropsychiatric disorders and constitute a potential treatment target. EMOTICOM is the first comprehensive neuropsychological test battery developed specifically to assess hot cognition. The aim of the study was to validate and establish a Danish language version and reference data for the EMOTICOM test battery. To evaluate the psychometric properties of 11 EMOTICOM tasks, we collected data from 100 healthy Danish participants (50 males, 50 females) including retest data from 49 participants. We assessed test–retest reliability, floor and ceiling effects, task-intercorrelations, and correlations between task performance and relevant demographic and descriptive factors. We found that test–retest reliability varied from poor to excellent while some tasks exhibited floor or ceiling effects. Intercorrelations among EMOTICOM task outcomes were low, indicating that the tasks capture different cognitive constructs. EMOTICOM task performance was largely independent of age, sex, education, and IQ as well as current mood, personality, and self-reported motivation and diligence during task completion. Overall, many of the EMOTICOM tasks were found to be useful and objective measures of hot cognition although select tasks may benefit from modifications to avoid floor and ceiling effects in healthy individuals.

## Introduction

Hot cognition describes cognitive processing of emotionally salient information (Roiser and Sahakian, 2013). Examples of hot cognitive domains include basic emotion processing, motivation and reward driven behaviors as well as social cognition, i.e., the ability to understand and participate in social transactions. Importantly, disruptions in hot cognitive processes have been identified as core features in a wide range of neuropsychiatric disorders such as mood disorders (Elliott et al., 2011), anxiety disorders (Plana et al., 2014), schizophrenia (Ventura et al., 2013), Attention Deficit and Hyperactivity Disorder (ADHD) (Umemoto et al., 2014), and autism (Harms et al., 2010). In particular, negative affective biases, i.e., the preferential processing of negative information over positive information, have consistently been shown in patients with mood disorders (Elliott et al., 2011; Hjordt et al., 2017), anxiety disorders (Mogg et al., 1995), substance abuse disorders (Ersche and Sahakian, 2007), and eating disorders (Lovell et al., 1997). Notably, one study found mood-congruent attentional biases in bipolar disorder where patients in the depressed state showed enhanced processing of negative information while patients in the manic state showed enhanced processing of positive information (García-Blanco et al., 2013). In contrast, healthy individuals typically show no or a slight positive affective bias (Pool et al., 2016). Meanwhile, impairments in motivation and reward-driven behaviors have been observed in psychopathological conditions including aggression (Kuin et al., 2015), traumatic brain injury (Newcombe et al., 2011), and ADHD (Umemoto et al., 2014) while differences in neural response to rewards and loss and disruptions in reinforcement learning have been linked to schizophrenia and major depressive disorder (MDD) (Chen et al., 2015; Hagele et al., 2015). Disturbances in social cognition including mentalization, i.e., the ability to infer the mental states of others, are central features of disorders such as autism and schizophrenia (Chung et al., 2014) and impairment in moral judgment has been reported for psychopathic individuals (Cardinale and Marsh, 2015), autism (Brewer et al., 2015), and patients suffering from ventromedial prefrontal cortex lesions (Cameron et al., 2018). In addition, self-blaming moral emotions such as guilt and shame have been shown to be exacerbated in MDD (Green et al., 2013) and anxiety disorders (Hedman et al., 2013). In healthy individuals, differences in hot cognitive processes have been linked to pharmacological interventions such as oxytocin (Leppanen et al., 2017) and serotonergic manipulations (Merens et al., 2007). Sub-clinical symptoms of depression and anxiety (Routledge et al., 2018), as well as natural sex hormone fluctuations in women (Osorio et al., 2018), also produce changes in hot cognition.

In summary, hot cognitive processes are relevant in a wide range of contexts across both normal and disturbed mental functioning. Notably, hot cognition has been proposed as an early predictor for treatment response in MDD (Harmer and Cowen, 2013; Park et al., 2018) as well as a promising target for therapeutic intervention (Roiser et al., 2012). Yet, despite growing recognition of their importance, scientists have so far lacked a validated and comprehensive set of tools capable of assessing hot cognitive processes in a standardized manner. Therefore, a group of researchers from Britain recently developed a novel 3-h computerized neuropsychological test battery called EMOTICOM (Bland et al., 2016). The EMOTICOM battery comprises 16 novel, adapted, and existing tasks designed to capture cognitive functions from four hot cognitive domains; (1) *Emotion Processing*, (2) *Motivation and Reward*, (3) *Impulsivity*, and (4) *Social Cognition*. The British developers validated the EMOTICOM battery in a cohort of 200 healthy participants (Bland et al., 2016). We here assess the psychometric properties of EMOTICOM in a shortened version using a Danish cohort of 100 healthy participants and provide reference data for research and clinical use of the test battery in Danish. In the British validation, test–retest reliability of the EMOTICOM battery was assessed after a relatively short time interval (5–10 days). In the present study we chose to collect retest data after 3–5 weeks in order to provide a reference for longitudinal studies investigating the effects of treatment or interventions over weeks or months. We also supplement the original British study findings by comparing performance on the EMOTICOM tasks in the shortened Danish battery with relevant factors such as personality, mood, and self-reported levels of motivation and diligence during task completion.

## Materials and Methods

### Participants

One hundred healthy Danish participants between 18 and 48 years of age (males, *n* = 50; females, *n* = 50) were recruited from a previously established database of healthy volunteers (Knudsen et al., 2016) or through internet advertisements and flyers posted around the greater Copenhagen area. Exclusion criteria for the study included history of psychiatric disorders, significant somatic illness, brain trauma, use of psychotropic medication, significant lifetime history of drug abuse, pregnancy or breastfeeding, and non-fluency in Danish. The study was approved by the Danish Data Protection Agency (protocol RH-2015-255) and written informed consent was obtained from all participants.

### Study Design

Upon inclusion, participants were randomized into single test or retest groups. Three participants originally randomized into the retest group dropped out after completing the first test session; one due to a family emergency and two failed to disclose the reason. To accommodate these dropouts, two unused single-test slots in the randomization system were converted into retest slots while the last dropout happened too late in the data collection process to be recovered. Thus, 51 participants completed a single test session while 49 participants completed retest sessions after 3–5 weeks (time between test–retest: 27.4 ± 4.8 days, mean ± SD)^1^. Intelligence quotient (IQ) was assessed with the Reynolds Intellectual Screening Test (RIST) using the verbal subtest ‘Guess What?’ and the non-verbal subtest ‘Odd-Item Out’ (Reynolds, 2011). Level of education was indexed with the Online Stimulant and Family History Assessment Module (OS-FHAM) questionnaire using a five-point Likert scale from 1 (no vocational degree) to 5 (>4 years of higher learning at university level). Personality was assessed with the NEO Personality Inventory Revised (NEO PI-R, *n* = 93) and the NEO Personality Inventory-3 (NEO PI-3, *n* = 6) (Costa and McCrae, 2005). Mood was assessed with the Profile of Mood State (POMS) (McNair and Heuchert, 2007) immediately before each test session. All test sessions took place in standardized testing rooms and were conducted by a team of five trained neuropsychological testers at the Neurobiology Research Unit, Copenhagen University Hospital Rigshospitalet.

In addition to a flat fee of 200 Danish kroners, participants had the opportunity to win money based on their performance in six EMOTICOM tasks that included monetary reward. For these six tasks, participants were instructed to rate their performance during the task in terms of motivation and diligence, i.e., the degree to which they had ‘done their best.’ Participants were also encouraged to write down any thoughts or suggestions regarding the overall test experience or any specific task, followed by a brief unstructured interview at the end of each session. The order of tasks within the EMOTICOM battery was randomized to control for any potential effects of test order.

### Translation and Implementation of EMOTICOM in Danish

Three native Danish speakers independently translated the full EMOTICOM test battery into Danish. Following a consensus meeting supervised by trained test psychologists, a single version was agreed upon. The consensus version was then back-translated into English by a natural English-Danish bilingual individual and sent for the approval of the original test developers. Implementation of the final Danish translation was done using the open source software PsychoPy. All monetary rewards were converted from British pounds to equivalent sums in Danish kroners.

### The EMOTICOM Test Battery

Out of the original 16 tasks in the full EMOTICOM test battery, 11 were selected for translation and implementation in the Danish version. Two tasks, *The Four-choice Serial Reaction Time Task* and *The Discounting Task*, were not translated into Danish because the original test code was unavailable while two others, *The Emotional Memory Recognition Task* and *The Inference Task*, were left out based on the recommendation from the original British test developers who felt these tasks warranted further improvements. Lastly, due to translation concerns (e.g., issues relating to word length, frequency, and translation ambiguity), the *Word Affective Go No/Go* was also not implemented in the Danish validation. Therefore, only three of the original four hot cognitive domains, i.e., *Emotion Processing*, *Reward and Motivation*, and *Social Cognition*, were represented in the present study, while the last domain, *Impulsivity*, was left out. For a brief overview of the selected EMOTICOM tasks and their primary outcomes see Table 1. For a description of the full EMOTICOM battery see Bland et al. (2016).

**TABLE 1:** EMOTICOM task overview.

**Emotion processing**	
Emotional Recognition Task	*Description*
	Assessment of emotion recognition. A series of emotional faces appear briefly (for 250 ms) and the participant is asked to identify the expressed emotion (happy, sad, angry, or fearful). The task has two versions: one using full faces and one showing only eyes.
	*Primary outcomes*
	Correct identification of each emotion calculated as hit rate (%).
Emotional Intensity Morphing Task	*Description*
	Assessment of perceptual threshold for emotion detection. A face with a slowly morphing emotional expression is shown. The participant must indicate when they can detect the presence of an emotion (increase condition) or no longer perceive an emotion (decrease condition). The emotional expressions include happy, sad, angry, fearful, and disgusted.
	*Primary outcomes*
	Intensity threshold for detection of each emotion in both the increase and decrease condition.
Face Affective Go/No-Go Task	*Description*
	Assessment of information processing bias in identification of emotional faces. A series of emotional faces (happy, sad, angry, or fearful) is shown and the participant is asked to respond only to a specific emotion while ignoring other emotions.
	*Primary outcomes*
	Discrimination accuracy of emotional faces indexed as *d*-prime scores for each emotion.
**Motivation and reward**	
Reinforcement Learning Task	*Description*
	Assessment of learning based on reward and punishment. A series of paired colored circles is shown and the participants is asked to choose one circle. Each color has either a high or low chance of eliciting a monetary reward (win condition) or a high or low risk of eliciting monetary loss (loss condition).
	*Primary outcomes*
	Learning rate (alpha) calculated with a reinforcement learning rate algorithm for both the no-loss and no-win condition.
Monetary Incentive Reward Task	*Description*
	Assessment of effort to avoid punishment and gain reward. The participant is asked to respond as quickly as possible when a black box appears between two circles each containing two lines. The distance between the lines indicate the size of the loss or gain for each trial. A faster response elicits greater reward/smaller loss.
	*Primary outcomes*
	Average change in reaction time relative to baseline reaction time for both the win and loss condition.
Progressive Ratio Task	*Description*
	Assessment of motivational breakpoint. Four boxes of varying sizes are shown and the participant is asked to select the odd one out. The frequency and size of monetary reward for successfully completing each trial is gradually decreased. The participant is told they can quit at any time but must still wait passively for the remainder of the task’s run time.
	*Primary outcomes*
	Motivational break-point, i.e., the number of trials the participant completes before quitting the task.
Adapted Cambridge Gambling Task	*Description*
	Assessment of decision making and risk-taking behavior. The participant is shown a roulette wheel divided into two colors; the proportion of each color changes in every trial, representing different odds. The participant is asked to choose the color they wish to bet on as well as the size of their bet. The task consists of a win and a loss condition.
	*Primary outcomes*
	Risk adjustment score indexing optimizing behavior in both the win and loss condition.
**Social cognition**	
Moral Emotions Task	*Description*
	Assessment of emotional reactions to moral social situations. The participant is presented with cartoons of moral scenarios in which one character intentionally or unintentionally harms another. The participant must rate how guilty, shameful, annoyed, and bad they would feel if they were either the victim or the agent (i.e., the victimizer).
	*Primary outcomes*
	Average ratings of guilt and shame for victim and agent scenarios.
Social Information Preference Task	*Description*
	Assessment of preference for different types of information. The participant is shown a socially ambiguous situation in which nine pieces of information (faces, thoughts, and facts/objects) are hidden from view. The participant is instructed to pick four pieces of information to help them decide between three different interpretations of the situations; a positive, neutral, and negative.
	*Primary outcomes*
	The proportion (%) of thoughts, faces, and facts chosen.
Prisoners’ Dilemma	*Description*
	Assessment of cooperative strategy. The participant and a computerized opponent perform a small task to collect money which is pooled. The participant is given the choice to split the money equally with the opponent or steal all the money. If both parties choose to split the money, both get half. If one steals and the other splits, the one who stole wins all the money. If both choose to steal, neither party wins any money. The participant faces three computerized opponents with different strategies: cooperative (opponent always splits), tit-for-two-tats (opponent splits until the participant steals for two consecutive trials), and aggressive (opponent starts with steal and then mirrors the participant’s behavior).
	*Primary outcomes*
	Proportion of trials (%) in which the participant chooses to steal for each type of opponent.
Ultimatum Game	*Description*
	Assessment of sensitivity to fairness. The participant and a computerized opponent perform a small task to collect money which is then pooled. In some trials, the participant decides how the money is split, ranging from fair (50/50) to increasingly unfair (10/90), and in some trials the opponent decides the split, ranging from fair (50/50) to increasingly unfair (10/90). The participants may choose to either accept or decline the offers from the opponent.
	*Primary outcomes*
	Proportion of accepted offers.

### Statistical Analysis

Statistical analyses were performed using SPSS statistical software (version 25.0) and R Studio (version 3.5). Missing data included NEO personality for one participant and self-reported ratings of motivation and diligence for five participants on the *Prisoner’s Dilemma* and for one participant on the *Ultimatum Game*. Alpha levels were set at 0.01 for statistical significance in order to account for multiple comparisons.

#### Task Outcomes and Descriptive Statistics

Primary task outcomes for each EMOTICOM task were selected based on recommendations from the original British test developers and the existing literature. Descriptive and psychometric information on secondary outcomes can be found in the Supplementary Information. Mean, SD, median, interquartile range, range, and skewness are reported for all primary task outcomes. Floor and ceiling effects were determined as the percentage of participants who achieved minimum scores (floor effect) or maximum scores (ceiling effects) for a given task outcome. Floor or ceiling effects above 10% were considered moderate while effects above 30% were considered severe/problematic.

#### Test–Retest Reliability

To assess test–retest reliability, intraclass correlation coefficients (ICCs) and their 95% confidence intervals (95% CI) were calculated based on retest data from 49 participants using an absolute-agreement two-way mixed effect model. ICC values of less than 0.40 were considered poor, values between 0.40 and 0.59 as fair, values between 0.60 and 0.74 as good, and values greater than 0.75 as excellent (Cicchetti, 1994). In addition, test–retest bias, i.e., percent change in scores between first and second test, was calculated as: *Test–retest bias* = *((score_*retest*_ – score_*test*_)/score_*test*_) ^∗^ 100*.

#### Task-Intercorrelations and Factor Analysis

To determine EMOTICOM’s ability to capture the three proposed underlying cognitive domains, correlation matrices conducted with Spearman’s rank correlations were used to index the shared marginal variance between tasks within the same cognitive domain, i.e., *Emotion Processing, Motivation and Reward*, and *Social Cognition*. In addition, we used an exploratory factor analysis to investigate the underlying factorial structure of the EMOTICOM test battery. The analysis was conducted using principal axis factoring with Varimax rotation. We used an eigen-value greater than 1 as criterion for extraction of factors.

#### Correlations With Demographic and Descriptive Factors

Spearman’s rank correlation was used to assess the association between performance on EMOTICOM tasks and relevant demographic and descriptive factors including age, sex, education, IQ, NEO personality trait Neuroticism, and scores for self-reported mood on test days. In addition, correlations between test performance and self-reported motivation and diligence were assessed for the six EMOTICOM tasks containing a monetary reward paradigm, i.e., *Reinforcement Learning Task, Monetary Incentive Reward Task, Progressive Ratio Task, Adapted Cambridge Gambling Task, Prisoner’s Dilemma*, and *Ultimatum Game*.

## Results

### Task Outcomes and Descriptive Statistics

Table 2 shows descriptive data for the 100 healthy Danish participants. Level of education was high with a majority (*n* = 74) of participants currently attending or having completed > 4 years of higher learning at university level. The study sample IQ of 110.36 was significantly higher than the population IQ of 100, *t*(99) = 14.8, *p* < 0.001 (Reynolds, 2011). There was no difference in Neuroticism scores between the study sample average of 76.04 and the Danish population average of 77.20, *t*(98) = −0.41, *p* = 0.68 (Skovdahl et al., 2011). Lastly, the study sample exhibited significantly lower levels of self-reported total mood disturbance (TMD) indexed with the POMS (TMD score = 1.56) compared to normative data (TMD score = 18.00), *t*(99) = −10.28, *p* < 0.001 (Nyenhuis et al., 1999).

**TABLE 2:** Descriptive data.

	***Mean***	***SD***	***Range***
Age (years)	28.87	7.33	18 to 48
Sex (male/female)	50/50		
Education (1–5)	4.54	0.58	1 to 5
IQ	110.36	6.98	93 to 129
Neuroticism^a^	76.04	27.89	24 to 144
TMD (−32 to 200)	1.56	15.99	−20 to 55

*Descriptive data for the Danish validation cohort (*N* = 100). The table shows age, education score indexed with the Online Stimulant and Family History Assessment Module on a five-point likert scale, IQ score assessed with the Reynolds Intellectual Screening Test, total mood disturbance (TMD) indexed with the Profile of Mood Scale, and trait Neuroticism indexed with the NEO Personality Inventory-Revised (*n* = 93) and the NEO Personality Inventory 3 (*n* = 6). ^*a*^*N* = 99 due to missing data from one participant.*

### Task Outcomes and Descriptive Statistics

Table 3 shows the descriptive statistics for the primary outcomes of each EMOTICOM task. A full overview of all secondary EMOTICOM outcomes can be found in Supplementary Information.

**TABLE 3:** Primary outcomes.

	***Mean***	***SD***	***Median***	***IQR***	***Range***	***Skewness***	***Floor effect***	***Ceiling effect***
**Emotional Face Recognition Task: Face version**								
Accuracy (%) – Happy	85.45	13.63	90.00	15.00	20 to 100	–2.42^∗∗∗^	0%	19%
Accuracy (%) – Sad	84.40	12.07	85.00	15.00	40 to 100	–1.31^∗∗∗^	0%	12%
Accuracy (%) – Angry	60.60	13.26	65.00	15.00	15 to 80	–1.27^∗∗∗^	0%	0%
Accuracy (%) – Fearful	82.00	11.87	85.00	15.00	45 to 100	–1.19^∗∗∗^	0%	6%
**Emotional Face Recognition Task: Eyes version**								
Accuracy (%) – Happy	78.15	16.46	80.00	20.00	20 to 100	–1.37^∗∗∗^	0%	6%
Accuracy (%) – Sad	71.20	19.06	75.00	25.00	10 to 100	–0.46^∗∗∗^	0%	5%
Accuracy (%) – Angry	66.20	11.81	65.00	20.00	40 to 90	–0.06^∗∗^	0%	0%
Accuracy (%) – Fearful	75.35	15.01	77.50	16.25	5 to 100	–0.41^∗∗∗^	0%	2%
**Emotional Intensity Morphing Task: Increase condition**								
Detection threshold – Happy	7.61	2.10	7.50	3.00	2.75 to 13.33	0.21	0%	0%
Detection threshold – Sad	9.46	2.13	9.50	3.00	3.50 to 13.50	–0.45	0%	0%
Detection threshold – Angry	8.79	2.18	8.71	2.31	3.50 to 14.00	0.11	0%	0%
Detection threshold – Fearful	9.58	2.33	9.50	3.25	4.00 to 15.00	–0.12	2%	0%
Detection threshold – Disgusted	9.06	2.06	9.50	2.75	3.50 to 13.50	–0.44	0%	0%
**Emotional Intensity Morphing Task: Decrease condition**								
Detection threshold – Happy	5.33	2.30	5.00	2.94	1.00 to 11.5	0.51^∗^	0%	6%
Detection threshold – Sad	5.47	1.73	5.50	2.19	1.75 to 10.25	0.29	0%	3%
Detection threshold – Angry	4.53	1.75	4.38	2.44	1.50 to 9.75	0.65^∗∗^	0%	7%
Detection threshold – Fearful	5.17	1.59	5.00	2.00	1.00 to 10.25	0.37	0%	3%
Detection threshold – Disgusted	4.04	1.75	3.75	2.31	1.00 to 10.50	0.85^∗∗^	0%	11%
**Face Affective Go/NoGo**								
*d*-prime – Happy/Neutral	2.85	0.67	2.93	0.73	−0.80 to 3.29	–2.70^∗∗∗^	0%	47%
*d*-prime – Happy/Sad	2.77	0.63	2.93	0.80	0 to 3.29	–1.60^∗∗∗^	0%	38%
*d*-prime – Neutral/Happy	2.50	0.81	2.93	0.76	0 to 3.29	–1.32^∗∗∗^	0%	19%
*d*-prime – Neutral/Sad	2.15	0.86	2.17	1.28	0 to 3.29	–0.63^∗∗∗^	0%	11%
*d*-prime – Sad/Happy	2.69	0.62	2.93	0.80	0.78 to 3.29	–1.23^∗∗∗^	0%	29%
*d*-prime – Sad/Neutral	2.05	1.01	2.17	1.28	−2.49 to 3.29	–1.61^∗∗∗^	0%	6%
**Reinforcement Learning Task^a^**								
Alpha – Win condition	0.23	0.33	0.02	0.40	0.00 to 1.00	1.33^∗∗∗^	32%	0%
Alpha – Loss condition	0.43	0.38	0.29	0.73	0.00 to 1.00	0.41^∗∗∗^	32%	0%
**Monetary Incentive Reward Task**								
Reaction time (ms) – Win condition	17.41	18.94	16.13	26.15	−30.3 to 72.87	0.05	–	–
Reaction time (ms) – Loss condition	18.73	18.45	16.67	25.88	−27.52 to 84.65	1.38	–	–
**Progressive Ratio Task**								
Breakpoint (trials)	316.77	148.33	424.50	251.00	1 to 436	–0.83^∗∗∗^	2%	48%
**Adapted Cambridge Gambling Task**								
Risk adjustment – Win condition	1.72	1.09	1.93	1.40	−0.56 to 3.56	–0.60^∗∗^	0%	0%
Risk adjustment – Loss condition	2.21	0.92	2.43	1.26	−0.71 to 3.64	–0.84^∗∗∗^	0%	0%
**Moral Emotions Task**								
Guilt – Agent	5.86	0.78	6.04	0.66	4.58 to 7.00	–2.08^∗∗∗^	0%	1%
Guilt – Victim	1.59	0.53	1.42	0.61	1.00 to 3.39	1.48^∗∗∗^	10%	0%
Shame – Agent	5.74	0.80	5.87	1.00	2.42 to 7.00	–1.35^∗∗∗^	0%	1%
Shame – Victim	1.97	0.70	1.91	1.00	1.00 to 4.42	0.78^∗∗^	8%	0%
**Social Information Preference Task**								
Information (%) – Thoughts	55.17	13.01	56.25	12.50	0.00 to 75.00	–1.64^∗∗∗^	1%	2%
Information (%) – Faces	11.52	11.38	7.81	10.16	0.00 to 57.81	1.83^∗∗∗^	5%	0%
Information (%) – Facts	33.31	9.34	32.81	10.94	7.81 to 57.81	–0.09	0%	0%
**Prisoner’s Dilemma**								
Proportion steals (%) – Cooperative	20.56	29.00	0.00	33.33	0 to 100	1.35^∗∗∗^	55%	5%
Proportion steals (%) – Tit-for-two-tat	25.56	32.84	0.00	52.78	0 to 100	0.89^∗∗∗^	54%	4%
Proportion steals (%) – Aggressive	35.00	32.03	33.33	66.67	0 to 100	0.3^∗∗∗^	33%	3%
**Ultimatum Game**								
Average acceptance rate (%)	61.07	26.64	59.52	42.26	14.29 to 100	0.01^∗∗∗^	0%	14%

*Mean, standard deviation (SD), median, interquartile range (IQR), range, and skewness are reported for the primary outcomes of the 11 EMOTICOM tasks. Shapiro–Wilks tests were used to assess normality of data; non-normal distribution of data is denoted with asterisks next to skewness (^∗^*p* < 0.05, ^∗∗^*p* < 0.01, ^∗∗∗^*p* < 0.001). Note, mean and SD should be used as reference for normally distributed outcomes while median and IQR should be used as reference for non-normally distributed outcomes. Floor and ceiling effects are presented as percentage of test subjects who achieved the minimum score (floor effect) or maximum score (ceiling effect). ^*a*^*N* = 68, as 32 participants performed below chance level, violating the assumptions of the reinforcement learning algorithm used to determine the alpha value.*

The majority of EMOTICOM task outcomes were skewed and 32 out of 42 outcomes had non-normal distributions. For these task outcomes, median and IQR should be used as reference instead of mean and SD. We observed small floor effects (<10%) for 4 outcomes; moderate floor effects (≥ 10%) for 1 outcome; and severe floor effects (≥30%) for 5 outcomes. In addition, we observed small ceiling effects for 15 EMOTICOM outcomes; moderate ceiling effects for 7 outcomes; and severe ceiling effects for 3 outcomes.

### Test–Retest Reliability

Table 4 shows test–retest reliability and test–retest bias for primary EMOTICOM outcomes.

**TABLE 4:** Test–retest reliability.

	**Baseline (*n* = 49)**		**Retest (*n* = 49)**		***Test–retest bias (%)***	***ICC***	***95% CI***
	***Mean***	***SD***	***Mean***	***SD***			
**Emotional Face Recognition Task: Face version**							
Accuracy (%) – Happy	85.92	13.76	90.20	13.38	4.98	0.83	0.66 to 0.91
Accuracy (%) – Sad	84.80	13.03	86.12	9.42	1.56	0.67	0.42 to 0.82
Accuracy (%) – Angry	63.27	12.73	70.00	12.42	10.64	0.60	0.25 to 0.78
Accuracy (%) – Fearful	83.47	10.62	83.27	9.77	–0.24	0.50	0.10 to 0.72
**Emotional Face Recognition Task: Eyes version**							
Accuracy (%) – Happy	80.41	14.21	80.51	14.44	0.12	0.50	0.10 to 0.72
Accuracy (%) – Sad	73.78	15.33	74.69	17.27	1.23	0.74	0.54 to 0.85
Accuracy (%) – Angry	69.49	10.96	74.29	11.90	6.91	0.65	0.36 to 0.80
Accuracy (%) – Fearful	77.86	12.20	79.08	11.02	1.57	0.64	0.36 to 0.80
**Emotional Intensity Morphing Task: Increase condition**							
Detection threshold – Happy	7.78	2.08	7.55	1.99	–2.96	0.67	0.48 to 0.80
Detection threshold – Sad	9.46	2.02	9.19	1.88	–2.85	0.57	0.35 to 0.73
Detection threshold – Angry	8.57	1.96	8.11	1.79	–5.37	0.66	0.41 to 0.81
Detection threshold – Fearful	9.33	1.98	9.04	2.2	–3.11	0.74	0.54 to 0.85
Detection threshold – Disgusted	9.05	2.04	8.21	1.78	–9.28	0.71	0.45 to 0.85
**Emotional Intensity Morphing Task: Decrease condition**							
Detection threshold – Happy	5.52	2.24	5.12	1.78	–7.25	0.75	0.56 to 0.86
Detection threshold – Sad	5.36	1.4	5.18	1.53	–3.36	0.50	0.11 to 0.72
Detection threshold – Angry	4.42	1.51	4.76	1.82	7.69	0.29	−0.25 to 0.60
Detection threshold – Fearful	5.03	1.25	4.65	1.42	–7.55	0.34	−0.14 to 0.63
Detection threshold – Disgusted	3.88	1.48	4.18	1.46	7.73	0.53	0.17 to 0.73
**Face Affective Go/NoGo**							
*d*-prime – Happy/Neutral	2.94	0.45	3.03	0.34	3.06	0.62	0.34 to 0.79
*d*-prime – Happy/Sad	2.79	0.69	2.88	0.44	3.23	0.12	−0.47 to 0.54
*d*-prime – Neutral/Happy	2.48	0.75	2.80	0.66	12.90	0.45	0.06 to 0.68
*d*-prime – Neutral/Sad	2.00	0.86	2.34	0.83	17.00	0.42	0 to 0.66
*d*-prime – Sad/Happy	2.79	0.55	2.73	0.57	–2.15	0.15	−0.52 to 0.52
*d*-prime – Sad/Neutral	2.15	0.84	2.43	0.81	13.02	0.44	0.03 to 0.68
**Reinforcement Learning Task^a^**							
Alpha – Win condition	0.23	0.33	0.21	0.37	–11.83	–0.04	−0.63 to 0.37
Alpha – Loss condition	0.46	0.36	0.43	0.41	–6.61	–0.18	−1.11 to 0.34
**Monetary Incentive Reward Task**							
Reaction time (ms) – Win condition	18.48	19.68	16.94	20.51	–8.34	–0.26	−1.07 to 0.25
Reaction time (ms) – Loss condition	19.05	20.86	18.98	21.33	–0.38	–1.47	−3.52 to −0.37
**Progressive Ratio Task**							
Breakpoint (trials)	309.76	153.64	350.69	130.61	13.21	0.56	0.24 to 0.75
**Adapted Cambridge Gambling Task**							
Risk adjustment – Win condition	1.78	1.17	2.36	0.82	32.58	0.20	−0.29 to 0.52
Risk adjustment – Loss condition	2.24	0.92	2.54	0.77	13.39	0.18	−0.40 to 0.53
**Moral Emotions Task**							
Guilt – Agent	5.88	0.79	5.85	0.69	–0.54	0.85	0.73 to 0.91
Guilt – Victim	1.63	0.54	1.69	0.51	3.49	0.83	0.70 to 0.90
Shame – Agent	5.80	0.82	5.68	0.73	–2.03	0.85	0.73 to 0.91
Shame – Victim	2.05	0.67	2.16	0.67	5.22	0.81	0.66 to 0.89
**Social Information Preference Task**							
Information (%) – Thoughts	51.5	15.43	53.99	14.73	4.83	0.71	0.48 to 0.83
Information (%) – Faces	13.52	13.19	14.00	13.55	3.55	0.74	0.54 to 0.85
Information (%) – Facts	34.98	9.90	32.02	6.90	–8.46	0.62	0.34 to 0.79
**Prisoner’s Dilemma**							
Proportion steals (%) – Cooperative	14.74	22.84	17.69	26.64	20.00	0.67	0.40 to 0.81
Proportion steals (%) – Tit-for-two-tat	18.82	28.62	16.55	23.69	–12.05	0.65	0.38 to 0.80
Proportion steals (%) – Aggressive	29.48	32.04	28.34	29.66	–3.85	0.67	0.42 to 0.82
**Ultimatum Game**							
Average acceptance rate (%)	59.14	25.05	71.96	27.68	21.69	0.77	0.46 to 0.89

*Test–retest reliability for the EMOTICOM tasks indexed with Intraclass Correlation Coefficients (ICC) and their 95% Confidence Intervals (95% CI). In addition, test–retest bias, i.e., percent change in scores between first and second test, was calculated as: *Test–retest bias* = *100 ^∗^ ((retest – test)/test).*^*a*^*N* = 35, as 14 participants performed below chance level, violating the assumptions of the reinforcement learning algorithm used to determine the alpha value.*

Intraclass correlation coefficients scores varied across primary EMOTICOM outcomes: 7 task outcomes exhibited excellent test–retest reliability (ICC ≥ 0.75); 21 task outcomes exhibited good test–retest reliability (0.60 ≤ ICC < 0.75); 9 task outcomes exhibited fair test–retest reliability (0.40 ≤ ICC < 0.60); and 10 outcomes exhibited poor test–retest reliability (ICC < 0.40). Test–retest bias ranged from −15.32 to 32.58% across all primary EMOTICOM outcomes.

### Task-Intercorrelations and Factor Analysis

**FIGURE 1:**
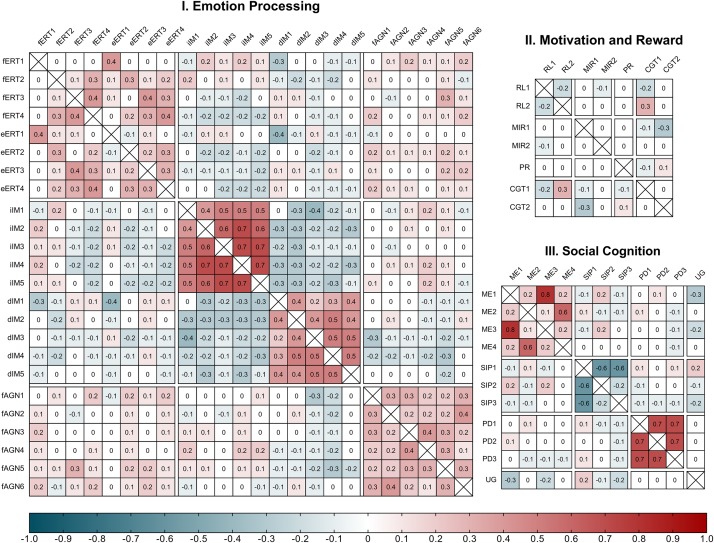
Spearman’s Rank Correlations for EMOTICOM outcomes within the three proposed cognitive domains. (I) Emotion Processing: fERT, face Emotion Recognition Task; fERT1, hit rate for happy; fERT2, hit rate for sad; fERT3, hit rate for angry; fERT4, hit rate for fearful. eERT, eyes Emotion Recognition Task; eERT1, hit rate for happy; eERT2, hit rate for sad; eERT3, hit rate for angry; eERT4, hit rate for fearful. iIM, increase Emotional Intensity Morphing Task; iIM1, detection threshold for happy; iIM2, detection threshold for sad; iIM3, detection threshold for angry; iIM4, detection threshold for fearful; iIM5, detection threshold for disgusted. dIM, decrease Intensity Morphing Task; dIM1, detection threshold for happy; dIM2, detection threshold for sad; dIM3, detection threshold for angry; dIM4, detection threshold for fearful; dIM5, detection threshold for disgusted. fAGN, Face Affective Go/NoGo Task; fAGN1, d-prime for ‘happy/neutral’; fAGN2, d-prime for ‘happy/sad’; fAGN3, d-prime for ‘neutral/happy’; fAGN4, d-prime for ‘neutral/sad’; fAGN5, d-prime for ‘sad/happy’; fAGN6, d-prime for ‘sad/neutral.’ (II) Motivation and Reward: RL, Reinforcement Learning Task; RL1, learning rate alpha for win condition; RL2, learning rate alpha for loss condition. MIR, Monetary Incentive Reward Task; MIR1, reaction time for win condition; MIR2, reaction time for loss condition. PR, Progressive Ratio Task. aCGT, adapted Cambridge Gambling Task; aCGT1, risk adjustment for win condition; aCGT2, risk adjustment for loss condition. (III) Social Cognition Domain: ME, Moral Emotions Task; ME1, guilt for agent; ME2, guilt for victim; ME3, shame for agent; ME4, shame for victim. SIP, Social Information Preference Task; SIP1, proportion thoughts; SIP2, proportion faces; SIP3, proportion facts. UG, Ultimatum Game.

Figure 1 shows the results of the correlation matrices conducted for each of the three cognitive domains: *Emotion Processing, Motivation and Reward*, and *Social Cognition*.

Within the *Emotion Processing* domain correlations between tasks were predominantly weak (−0.2 < ρ < 0.2) and statistically non-significant at the 0.01 alpha level. Only three pairs of task outcomes showed statistically significant correlations: accuracy for Anger in the *face Emotional Recognition Task* and d-prime for Happy/Neutral in the *Face Affective Go/NoGo task* (ρ = 0.30, *p* = 0.003); accuracy for Happy in the *eyes Emotional Recognition Task* and detection threshold for Happy in the decrease condition of the *Emotional Intensity Morphing task* (ρ = −0.36, *p* < 0.001); and detection threshold for Anger in the decrease condition of the *Emotional Intensity Morphing task* and d-prime for Happy/Neutral in the *Face Affective Go/NoGo task* (ρ = −0.31, *p* = 0.002). Meanwhile correlations between outcomes within the same task ranged from week to moderate for the *Emotional Recognition Task* (ρ = [−0.12;0.45]); from weak to strong for the *Emotional Intensity Morphing task* (ρ = [−0.35;0.70]); and from weak to moderate for the *Face Affective Go/NoGo task* (ρ = [0.13;0.35]). Within the *Motivation and Reward* domain correlations between tasks were predominantly weak (−0.2 < ρ < 0.2) and statistically non-significant at the 0.01 alpha level. Only one pair of outcomes showed a statistically significant correlation: reaction time for the win condition in the *Monetary Incentive Reward task* and risk adjustment for the win condition in the *Adapted Cambridge Gambling Task* (ρ = −0.28, *p* = 0.005). Correlations between outcomes within the same task was moderate for the *Reinforcement Learning Task* (ρ = −0.22); weak for the *Monetary Incentive Reward task* (ρ = 0.05); and weak for the *Adapted Cambridge Gambling Task* (ρ = 0.04). Within the *Social Cognition Domain* correlations between tasks were predominantly weak (−0.2 < ρ < 0.2) and statistically non-significant. Only one pair of outcomes showed a statistically significant correlation: Agent Guilt rating from the *Moral Emotions* task and average acceptance rate from the *Ultimatum Game* (ρ = −0.28, *p* = 0.006). Correlations between outcomes within the same task ranged from weak to strong for the *Moral Emotions task* (ρ = [0.13;0.76]); from weak to strong for the *Social Information Preference task* (ρ = [−0.61; −0.17]); and were strong for the *Prisoner’s Dilemma* task (ρ = [0.67;0.71]).

The exploratory factor analysis indicated a 13-factor solution with a majority of factors loading onto a single task (see Supplementary Information for summary of factor loadings). The 13 factors cumulatively accounted for 70.4% of the total variance. The Kaiser-Meyer-Olkin measure of sampling adequacy was low but acceptable (KMO = 0.53) and Bartlett’s test of sphericity was significant [χ^2^(820) = 1807.0, *p* < 0.001], indicating that the data was suitable for structure detection.

### Correlations With Demographic and Descriptive Factors

Table 5 shows correlations between primary EMOTICOM outcomes and various demographic and descriptive factors. A full overview of correlation between demographic and descriptive factors and all EMOTICOM outcomes can be found in Supplementary Information.

**TABLE 5:** Correlations.

	***Age***	***Sex*^$^**	***Education***	***IQ***	***Neuroticism*^a^**	***TMD***	***Motivation***	***Diligence***
**Emotional Face Recognition Task: Face version**								
Accuracy (%) – Happy	–0.04	0.01	–0.10	0.04	0.00	0.17	–	–
Accuracy (%) – Sad	–0.19	–0.15	0.05	0.15	0.19	0.28^∗∗^	–	–
Accuracy (%) – Angry	–0.32^∗∗∗^	–0.02	–0.05	0.16	0.14	–0.05	–	–
Accuracy (%) – Fearful	–0.38^∗∗∗^	0.09	–0.01	0.16	0.14	0.19	–	–
**Emotional Face Recognition Task: Eyes version**								
Accuracy (%) – Happy	–0.01	0.06	–0.21	0.09	0.04	0.14	–	–
Accuracy (%) – Sad	–0.17	0.18	–0.02	–0.01	0.23	0.21	–	–
Accuracy (%) – Angry	–0.03	0.07	–0.04	–0.004	0.14	0.07	–	–
Accuracy (%) – Fearful	–0.24	0.02	–0.04	–0.003	0.14	0.04	–	–
**Intensity Morphing Task: Increase condition**								
Detection threshold – Happy	–0.03	–0.01	0.07	0.06	0.06	0.05	–	–
Detection threshold – Sad	0.04	–0.09	0.08	0.12	–0.05	–0.05	–	–
Detection threshold – Angry	–0.02	–0.12	0.12	0.07	0.03	0.09	–	–
Detection threshold – Fearful	0.15	–0.12	0.15	0.06	–0.13	0.04	–	–
Detection threshold – Disgusted	0.12	–0.19	0.14	0.09	–0.05	–0.04	–	–
**Intensity Morphing Task: Decrease condition**								
Detection threshold – Happy	–0.08	0.00	0.02	–0.10	–0.02	–0.06	–	–
Detection threshold – Sad	–0.03	0.03	–0.15	–0.21	–0.05	–0.13	–	–
Detection threshold – Angry	–0.03	–0.18	–0.12	0.02	–0.01	0.00	–	–
Detection threshold – Fearful	0.08	0.05	–0.27^∗∗^	–0.19	–0.03	0.00	–	–
Detection threshold – Disgusted	–0.02	–0.09	–0.06	0.01	–0.07	–0.11	–	–
**Face Affective Go/NoGo**								
d-prime – Happy/Neutral	0.02	0.10	0.04	0.05	–0.05	–0.07	–	–
d-prime – Happy/Sad	0.05	0.08	–0.08	0.04	–0.01	0.03	–	–
d-prime – Neutral/Happy	0.04	0.001	0.05	–0.07	0.05	0.05	–	–
d-prime – Neutral/Sad	0.02	–0.09	0.14	0.11	0.06	0.22	–	–
d-prime – Sad/Happy	0.00	–0.10	0.09	0.05	–0.04	0.01	–	–
d-prime – Sad/Neutral	–0.08	0.21	–0.19	–0.03	–0.02	0.14	–	–
**Reinforcement Learning Task^b^**								
Alpha – Win condition	–0.30	0.13	–0.04	–0.23	0.29	0.06	0.05	0.01
Alpha – Loss condition	0.23	–0.16	0.14	0.05	–0.31	0.03	–0.13	–0.20
**Monetary Incentive Reward Task**								
Reaction time (ms) – Win	–0.08	–0.01	0.14	–0.11	0.15	–0.18	–0.08	0.09
Reaction time (ms) – Loss	0.02	0.06	–0.07	–0.10	–0.02	–0.14	–0.06	0.09
**Progressive Ratio Task**								
Breakpoint (trials)	–0.23	0.12	0.05	–0.09	–0.07	–0.07	0.39^∗∗∗^	0.29^∗∗^
**Adapted Cambridge Gambling Task**								
Risk adjustment – Win condition	0.12	–0.28^∗∗^	0.11	0.19	0.05	0.05	–0.16	–0.05
Risk adjustment – Loss condition	–0.21	0.03	0.17	0.14	–0.01	–0.01	0.02	0.06
**Moral Emotions Task**								
Guilt – Agent	0.14	0.17	–0.05	–0.11	0.01	0.01	–	–
Guilt – Victim	0.08	0.17	–0.07	–0.03	0.15	0.15	–	–
Shame – Agent	0.02	0.28^∗∗^	–0.02	–0.17	0.1	0.10	–	–
Shame – Victim	–0.03	0.16	–0.03	–0.17	0.23	0.23	–	–
**Social Information Preference Task**								
Information (%) – Thoughts	–0.02	0.03	0.02	–0.05	–0.1	–0.10	–	–
Information (%) – Faces	0.13	0.03	–0.10	0.03	0.06	0.06	–	–
Information (%) – Facts	–0.08	–0.09	0.10	0.10	0.07	0.07	–	–
**Prisoner’s Dilemma^c^**								
Proportion steals (%) – Cooperative	–0.13	–0.14	–0.02	–0.07	–0.12	–0.12	0.01	0.06
Proportion steals (%) – Tit-for-two-tat	–0.08	–0.23	–0.01	–0.11	–0.01	–0.01	0.04	0.10
Proportion steals (%) – Aggressive	–0.06	–0.26^∗∗^	0.03	0.005	–0.03	–0.03	0.11	0.14
**Ultimatum Game^d^**								
Average acceptance rate (%)	–0.16	0.07	0.16	–0.08	0.06	0.17	–0.02	–0.22

*Correlations between EMOTICOM primary outcomes and age, sex, education indexed with the Family History Assessment Module on a five-point Likert scale, IQ score assessed with the Reynolds Intellectual Screening Test, total mood disturbance (TMD) indexed with the Profile of Mood Scale, and trait Neuroticism indexed with the NEO Personality Inventory-Revised (*n* = 93) and the NEO Personality Inventory 3 (*n* = 6). Correlations between self-reported motivation and diligence and outcomes from the six EMOTICOM tasks containing monetary reward are also shown. Correlation coefficients are reported as Spearman’s ρ; only *p*-values < 0.01 are considered significant. ^∗∗^*p* < 0.01, ^∗∗∗^*p* < 0.001. ^$^A negative ρ value indicates males score higher while a positive ρ value indicates females score higher. ^*a*^*N* = 99 due to missing data from one participant. ^*b*^*N* = 68 as 32 participants performed below chance level, violating the assumptions of the reinforcement learning algorithm used to determine the alpha value. ^*c*^*N* = 95 due to missing data from five participants. ^*d*^*N* = 99 due to missing data from one participant.*

Age was negatively correlated with accuracy in recognizing angry and fearful emotions in the eyes version of the *Emotional Face Recognition Task* while differences in sex were correlated with risk adjustment in the win condition in the *Adapted Cambridge Gambling Task* (men performed better); ratings of shame in the *Moral Emotions task* (women rated higher); and proportion of steals against and aggressive opponent in the *Prisoner’s Dilemma* (men stole more). Education level showed a negative correlation with detection threshold of fearful emotions in the decrease condition of *Intensity Morphing task* while IQ and Neuroticism scores were not statistically correlated with performance on any primary outcome. Negative mood was positively correlated with accuracy in recognizing sad emotions in the face version of the *Emotional Face Recognition Task* and self-rated motivation and diligence during task completion was positively correlated with breakpoint in the *Progressive Ratio Task*.

## Discussion

We here present data collected from 100 healthy participants in order to validate the EMOTICOM test battery and provide reference material for future clinical and research use in Danish populations. Overall the shortened EMOTICOM test battery exhibited mostly acceptable test–retest reliability, low task-intercorrelations indicating limited redundancy between the tasks, and independence between task performance and demographic factors. Therefore, many of the EMOTICOM tasks provide a useful objective method for measuring hot cognition. Below we discuss some task-specific considerations regarding the use of the EMOTICOM test battery in research or clinical practice.

### Skewness of Data

A majority of primary EMOTICOM outcomes (76%) exhibited non-normal distributions. One explanation for this could be that our study sample is biased or that the tasks contain threshold constraints such as floor or ceiling effects which skew the distribution. The observed non-normal distributions may also reflect that the construct being assessed is not normally distributed within the general population. For example, norm data reported for emotion recognition paradigms similar to those included in the EMOTICOM test battery indicate that the performance of healthy individuals is not normally distributed within this cognitive domain (Kessels et al., 2014). Due to the skewness observed in some of the EMOTICOM tasks, we recommend using the median and interquartile ranges to gauge task performance instead of mean and SD.

### Floor and Ceiling Effects

Floor and ceiling effects occur when a task is either too difficult (floor effect) or too easy (ceiling effect). It represents a serious psychometric issue because it limits the variability of the collected data and therefore the amount of useful information obtained. Several EMOTICOM tasks exhibited floor or ceiling effects: out of the 42 primary task outcomes, 16 outcomes exhibited either floor or ceiling effects above 10% (i.e., at least 10% of all participants achieved either minimum or maximum scores), including eight outcomes that exhibited severe floor or ceiling effects of 30–55%. In particular, the *Face Affective Go/NoGo Task* had severe ceiling effects while the *Reinforcement Learning Task* had severe floor effects. For the *Face Affective Go/NoGo Task*, this issue could potentially be helped by using reaction time instead of *d*-prime as the primary outcome as reaction time is less vulnerable to floor and ceiling effects. Meanwhile, the presence of floor effects was particularly problematic for the *Reinforcement Learning Task* as a basic assumption in the algorithm used to determine the main outcome (learning rate, alpha) is that the participant performs better than chance level, i.e., that they learn the rules for choosing the best option and stop guessing randomly. In the present sample this meant that the learning rate could not be computed for 32 of the 100 participants. The difficulty of the task was corroborated by the unstructured interviews in which many participants reported they were unable to detect any patterns and kept randomly guessing throughout the task. We therefore suggest that the *Reinforcement Learning Task* may benefit from modifications or at least careful consideration before being applied in clinical practice or research. Other tasks including the *Prisoner’s Dilemma Task* and the *Progressive Ratio Task* also had a large proportion of participants who met our criteria for ceiling effects. However, as the purpose of these tasks is to assess different behavioral strategies (e.g., aggressive vs. cooperative) we argue that it is not meaningful to use the terms floor and ceiling effects in the conventional sense for these types of tasks even though they contain optimal strategies for maximizing monetary reward (e.g., not quitting in the *Progressive Ratio Task*).

### Test–Retest Reliability

In the original British validation study, test–retest reliability was assessed over a time-period of 5–10 days while we chose a retest span of 3–5 weeks. This longer timeframe is suited to inform studies that include long-term interventions or follow clinical progress over time. However, life events and mood may change considerably more over periods of weeks, as compared with days, which may influence test–retest reliability. The majority of EMOTICOM task outcomes exhibited fair to excellent test–retest reliability although notably only two tasks, the *Moral Emotions task* and the *Ultimatum Game*, had excellent test–retest coefficients of ≥ 0.75 for all primary outcomes. In addition, several tasks showed very poor reliability including the *Face Affective Go/NoGo Task*, *Monetary Incentive Reward Task*, and the *Adapted Cambridge Gambling Task*. It should be noted that low ICC scores can be caused by limited variance in the data which in turn may occur as a result of ceiling or floor effects (Koo and Li, 2016). For example, the low ICC scores reported for the *Face Affective Go/NoGo Task* may in part be explained by the severe ceiling effects exhibited by this task. Overall, tasks from the *Social Cognition* domain appeared to have the highest degree of reliability followed by tasks from the *Emotional Processing* domain, while tasks from the *Motivation and Reward* domain had poorer reliability. These observations were largely in accordance with the reports from the original British validation study for related outcomes from the same tasks (Bland et al., 2016). However, what may appear as poor reliability for *Motivation and Reward* tasks could instead reflect learning effects or adaptation in playing strategy. For instance, several participants reported deliberately prioritizing optimizing their winnings during their second session rather than ‘playing fair’ against the computer opponent. Furthermore, the reported test–retest biases were predominantly positive across most tasks, supporting the presence of a slight behavioral learning effect. It should be noted that for tasks without right/wrong answers (e.g., *Moral Emotions Task* and *Prisoner’s Dilemma*), the test–retest bias cannot be interpreted as a learning effect but could instead reflect a shift in response style or choice of strategy.

### Construct Validity

The tasks in the EMOTICOM test battery were originally chosen to capture distinct hot cognitive domains including *Emotion Processing*, *Motivation and Reward*, and *Social Cognition*. In order to test the extent to which each individual task loaded onto their respective domains, we mapped the shared variance for the task outcomes within the same domain in three correlation matrices. We found that there were little to no correlation between tasks from the same hot cognitive domain indicating that the original hypothesis of task specific domains could not be supported. This was further corroborated by the results of the exploratory factor analysis which indicated a 13-factor solution and thus did not support the proposed three-domain factorial structure. These results align with the findings from the original British validation which also failed to detect the proposed domain-specific pattern across EMOTICOM tasks (Bland et al., 2016). A possible explanation is that the proposed hot cognitive domains do not represent a single unitary cognitive construct; instead they should be seen as umbrella-terms for multiple inter-related cognitive processes. In addition, while previous studies have indicated the existence of an underlying facial expression decoding construct in the *Emotion Processing domain* (Hildebrandt et al., 2015), we speculate that the EMOTICOM tasks within this domain are too heterogeneous both in terms of task design and outcome scales to capture this single construct. Overall, these findings emphasize that hot cognition is a complex phenomenon made up of multifaceted cognitive constructs. As a consequence, we recommend that researchers aiming to investigate hot cognition using EMOTICOM should view the battery as a tool box and carefully consider the exact target of their investigation before choosing the appropriate task.

Lastly, some EMOTICOM tasks exhibited very low within-task correlation, suggesting that (a) the task itself does not measure a single construct or (b) the outcomes are unreliable. This was particularly pronounced for tasks from the *Motivation and Reward* domain and indicates that these tasks may benefit from modifications.

### Demographic Factors

With few exceptions, performance on EMOTICOM tasks was not strongly influenced by demographic factors. Age was negatively correlated to recognition of anger and fear in the face version of the *Emotional Face Recognition Task* but not in the eye version. Age effects on emotion recognition have previously been reported in the literature and in particular for recognition of negative emotions (Ruffman et al., 2008). Therefore, it may be advantageous to use the eye version of the *Emotional Face Recognition Task* in study cohorts containing middle-aged and older adults as this version appears to be less sensitive to age effects. Corroborating the original British validation study, we did not observe sex effect on tasks from the *Emotion Processing* domain (Bland et al., 2016), but women exhibited higher ratings of shame in the *Moral Emotions Task*. This fits with previous reports of sex differences in proneness to experience shame and guilt (O’Connor et al., 1994; Else-Quest et al., 2012). Women were also less likely to steal from their opponent in the *Prisoner’s Dilemma* task while men exhibited better risk adjustment in the *Adapted Cambridge Gambling Task*. Performance on EMOTICOM appeared to be largely independent of IQ and education with the single exception of a negative correlation between education level and detection of fear in the *Intensity Morphing task*’s decrease condition. However, it should be emphasized that the included participants were not stratified for education. This resulted in a cohort with very high education levels as well as high IQ which limits our ability to accurately assess the potential effect of these factors on task performance. Overall, it is a strength of the EMOTICOM test battery that demographic factors do not seem to influence task performance. However, given the stratification issues described above, other studies are needed to investigate the impact of demographic factors on test performance in older as well as less well-educated cohorts.

### Mood, Personality, Motivation Factors

In addition to demographic characteristics, we also looked at how other relevant factors such as trait Neuroticism and self-reported mood might influence responses on EMOTICOM tasks. Trait Neuroticism is used to index the tendency to experience negative emotions and is strongly linked to risk of developing psychopathology (Malouff et al., 2005; Ormel et al., 2013). Trait Neuroticism did not correlate significantly with any EMOTICOM outcomes while mood was positively correlated with recognition of sad faces in the face version of the *Emotional Face Recognition Task* only. The latter finding is in line with previous reports showing that mood can influence recognition of emotional faces. However, the effect appears to be relatively small and in most studies requires the active evocation of emotion in the participant prior to the presentation of the stimuli (Schmid and Mast, 2010). Lastly, the correlation between self-reported motivation and diligence during the six tasks containing the possibility of winning an extra sum of money was also assessed. We found that self-reported motivation and diligence had little effect on performance except for motivation on the *Progressive Ratio Task.* This provides further validation for the Progressive Ratio Task as an objective measure of motivation. Overall, the general lack of correlations between performance on EMOTICOM tasks and trait Neuroticism, mood disturbance, and self-reported motivation and diligence indicates that EMOTICOM is not sensitive to differences in emotion fluctuations or personality characteristics in healthy participants.

### Comparison With British Validation Study

There are several differences between the original British validation study and the present work. For example, we chose a longer test–retest interval and included measures of mood, Neuroticism and motivation and diligence to characterize potential influences on task performance. In addition, many of the reported task outcomes differ. We based our choice of primary outcomes for each task on consultation with the original test developers as well as standard practice in the literature. However, as most cognitive tasks do not have a single, clearly defined outcome, the ‘optimal’ choice of primary outcome may vary from study to study depending on the research question. For example, recognition of angry faces may be especially relevant in studies investigating aggression whereas recognition of fearful faces may be especially relevant for studying anxiety. We therefore endeavored to pick outcomes that we believe best capture the core cognitive function of each task and, when possible, limit the use of composite outcomes (i.e., complex outcomes created from two or more outcomes). While these choices make a direct one-to-one comparison between the two studies difficult, overall our findings align with those from the British validation study. We observed similar patterns of test–retest reliability at both task and domain level and were able to replicate the report that EMOTICOM is largely independent of demographic factors. In addition, we corroborate the original study’s rejection of a three-domain structure. As information on floor and ceiling effects were not reported in the British validation study, we cannot compare our results to the British study.

### Methodological Limitations

EMOTICOM was initially validated in 200 volunteers by the British test developers. The purpose of this study was to replicate the original study with a smaller sample of 100 Danish participants. This is a used practice for psychometric studies comparing populations with large biological, environmental, and cultural overlaps; e.g., the Danish version of the Delis-Kaplan Executive Function System (D-KEFS) test battery was validated against American norms based on data collected from 111 Danish individuals. However, the relatively small sample size of the present study does present some limitations. In particular the reported correlations between task performance and demographic and descriptive factors should be interpreted with caution as the study may not have had sufficient power to detect weaker correlations. In addition, as the present study likely does not have a sufficiently large sample size to accurately estimate the true factorial structure of the EMOTICOM task outcomes (Beavers et al., 2013), we refrain from interpreting the meaning of individual factors derived from the analysis. Importantly, our study sample does not represent a normative sample but rather a reference sample based on well-educated individuals with high IQ. In addition, due to the high level of ethnic and cultural homogeneity in the Danish population, the present study sample could not provide any insight into potential effects of ethnicity or cultural differences on task performance. Therefore, caution should be taken when comparing the findings to other types of study groups or the general population. Also, based on the current study it cannot be ascertained whether the observed ceiling effects in healthy participants would also be present in clinical samples nor how sensitive the tasks may be to psychological or pharmacological interventions. So far, one study has used the EMOTICOM battery to investigate the association between paranoid thinking in healthy participants and social cognition, reporting a link between increased paranoia and likelihood of stealing from the cooperative opponent in the *Prisoner’s Dilemma* task (Savulich et al., 2018).

As a final note, we caution against using the rating of ‘annoyance’ from the *Moral Emotions* task. Based on the qualitative interviews, we discovered that some participants reported high levels of annoyance in moral scenarios where they were the agent (i.e., when they caused harm to others) because they ‘felt annoyed with themselves’ while some participants reported low levels of annoyance because they ‘did not feel annoyed with the victim or the situation.’ Since this ambiguity of interpretation was not seen in the original publication of a healthy United Kingdom sample, it may reflect cultural differences. We therefore recommend that the task instructions be modified to eliminate this ambiguity.

## Conclusion

We here present reference material for performance on the hot cognitive test battery EMOTICOM from a Danish cohort of healthy participants. While most tasks exhibited acceptable psychometric properties, select tasks may not be appropriate for use in healthy individuals due to issues relating to floor and ceiling effects, low test–retest reliability and lack of within-task correlations. While these issues may be ameliorated by choosing alternate task outcomes in some cases (e.g., for the *Face Affective Go/NoGo* task) other tasks, in particular those from the *Motivation and Reward* domain, may benefit from modifications. We observed overall weak correlations between tasks within the same domain, indicating that the proposed structure of an *Emotion Processing* domain, *Reward and Motivation* domain and *Social Cognition* domain cannot be substantiated. EMOTICOM tasks were largely independent of demographic factors such as age, sex, education as well as IQ, personality, mood, and self-reported motivation and diligence during task completion. The present study may help guide future study designs by indicating which EMOTICOM tasks may be most appropriate for the study population planned. In conclusion, many EMOTICOM tasks provide useful, objective methods for measuring social and emotional cognition; however, future studies are needed to investigate the performance of EMOTICOM tasks in patient groups as well as their performance in intervention trials.

## Data Availability Statement

For legal reasons we are not allowed to upload and share our data. The data from the study is available upon request from the CIMBI database (http://www.cimbi.dk/db).

## Ethics Statement

Ethical review and approval was not required for the study on human participants in accordance with the local legislation and institutional requirements. The patients/participants provided their written informed consent to participate in this study.

## Author Contributions

DS, VF, and GK conceived and designed the study. VD and CT collected the data. PJ organized the database. VD, PJ, and AB defined and implemented the outcomes used. VD wrote the first draft of the manuscript. EM consulted on the statistical analysis which was performed by VD. CT wrote the sections of the manuscript. RE and BS consulted on the analysis and interpretation of the findings. All authors contributed to the manuscript revision, and read and approved the submitted version.

## Conflict of Interest

AB, RE, and BS are co-inventors of the EMOTICOM test battery and BS consults for Cambridge Cognition. The remaining authors declare that the research was conducted in the absence of any commercial or financial relationships that could be construed as a potential conflict of interest.

## Footnotes

1 Due to scheduling conflicts, one participant completed the retest session after 6 weeks (43 days).
