# Supplementary material for: Psychometric Properties and Validation of the EMOTICOM Test Battery in a Healthy Danish Population
Source: Front Psychol. 2019 Dec 3;10:2660. doi: 10.3389/fpsyg.2019.02660 (PMC6901831; doi:10.3389/fpsyg.2019.02660)

# Appendix


## Emotion Processing

1. Emotional Recognition Task…………………………………………………………… p. 2
2. Emotional Intensity Morphing Task………………………………………………... p. 6
3. Face Affective Go/NoGo Task…………………………………………………….... p. 7

## Motivation and Reward

4. Reinforcement Learning Task……………………………………………………… p. 11
5. Monetary Incentive Reward Task………………………………………………….. p. 14
6. Progressive Ratio Task……………………………………………………………….. p. 17
7. Adapted Cambridge Gambling Task………………………………………………. p. 20

## Social Cognition

8. Moral Emotions Task……………………………………………………………… p. 24
9. Social Information Preference Task……………………………………………….. p. 28
10. Prisoner's Dilemma………………………………………………………………... p. 31
11. Ultimatum Game…………………………………………………………………... p. 35

**Exploratory factor analysis**…………………………………………………………… p. 39

# 1. Emotional Recognition Task

**Task description**

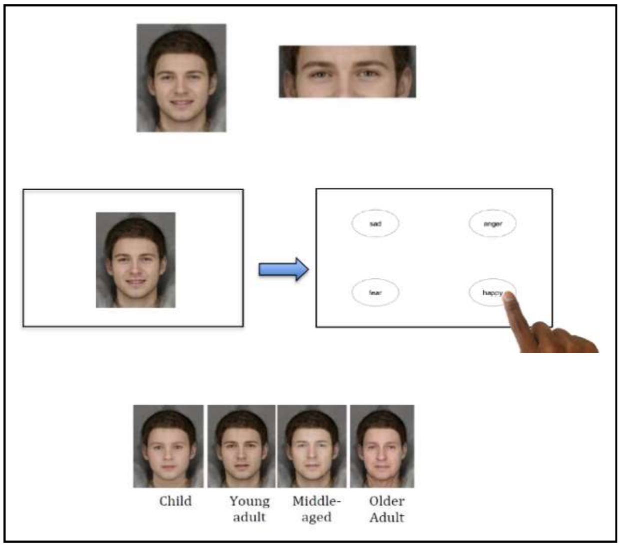


The face and eyes Emotional Recognition Task (fERT & eERT) measures the ability to identify emotions in facial expressions. The EMOTICOM test battery contains two separate ERT task versions: a full-face version and an eyes-only version. The faces, or eyes, are briefly presented on a computer screen (250ms) after which the test subject is asked to identify the emotion expressed (happy, sad, angry, or fearful). Each emotion is shown twice at ten different intensity levels (10%, 20%, 30%, 40%, 50%, 60%, 70%, 80%, 90%, 100%) equaling 20 trials for each emotion and 80 trials in total. In addition, a control condition displaying faces/eyes at different ages (child, young adult, middle aged, and older adult) is also included in the task.

**Main outcomes** (reported in the main article)

- *Hit rate (H)* – Percentage of trials in which a given emotion is correctly identified

**Secondary outcomes**

- *False alarm rate (FA)* – Percentage of trials in which a given emotion is incorrectly identified
- *d-prime (d')* – Index of discrimination sensitivity calculated as: $d' = z(H) - z(FA)$.

**Emotional Recognition Task – Descriptive data**

| | *Mean* | *SD* | *Median* | *IQR* | *Range* | *Skewness* | *Floor effect* | *Ceiling effect* |
|---|---|---|---|---|---|---|---|---|
| **Face version** | | | | | | | | |
| False alarm rate (%) - Happy | *8.17* | *8.82* | 5.00 | 10.00 | 0.00–40.00 | 1.49 | 0% | 15% |
| False alarm rate (%) - Sad | *15.42* | *8.48* | 15.00 | 8.75 | 0.00–46.67 | 0.89 | 0% | 2% |
| False alarm rate (%) - Angry | *1.83* | *3.59* | 0.00 | 1.67 | 0.00–21.67 | 3.65 | 0% | 53% |
| False alarm rate (%) - Fearful | *3.77* | *4.15* | 1.67 | 6.67 | 0.00–20.00 | 1.55 | 0% | 28% |
| *d*-prime - Happy | *2.76* | *0.52* | 2.80 | 0.61 | 1.09–3.92 | -0.53 | 0% | 0% |
| *d*-prime - Sad | 2.21 | 0.56 | 2.24 | 0.71 | 0.42–3.46 | -0.56 | 0% | 0% |
| *d*-prime - Angry | *2.16* | *0.42* | 2.21 | 0.52 | 0.46–2.8 | -1.02 | 0% | 0% |
| *d*-prime - Fearful | *2.76* | *0.52* | 2.80 | 0.61 | 1.09–3.79 | -0.48 | 0% | 0% |
| **Eyes version** | | | | | | | | |
| False alarm rate (%) - Happy | *15.00* | *12.06* | 11.67 | 13.33 | 0–63.33 | 1.43 | 0% | 3% |
| False alarm rate (%) - Sad | *15.18* | *8.49* | 13.33 | 11.67 | 1.67–41.67 | 0.87 | 0% | 0% |
| False alarm rate (%) - Angry | *3.63* | *5.94* | 1.67 | 5.00 | 0–50 | 5.24 | 0% | 32% |
| False alarm rate (%) - Fearful | *2.55* | *3.90* | 1.67 | 3.33 | 0–26.67 | 3.14 | 0% | 45% |
| *d*-prime - Happy | 2.59 | 0.57 | 2.63 | 0.66 | 0.32–3.92 | -0.49 | 0% | 0% |
| *d*-prime - Sad | 1.75 | 0.59 | 1.77 | 0.75 | 0.48–3.29 | -0.12 | 0% | 0% |
| *d*-prime - Angry | 2.22 | 0.40 | 2.21 | 0.46 | 1.04–3.24 | -0.09 | 0% | 0% |
| *d*-prime - Fearful | *2.59* | *0.57* | 2.63 | 0.66 | 0.32–3.92 | -0.89 | 0% | 0% |

**Legend:** Mean, SD, median, interquartile range, range, and skewness are shown. Note, for outcomes with non-parametric data distribution, mean and SD are denoted in cursive gray indicating the median and interquartile range should be used as reference. Floor and ceiling effects are shown as percentage of test subjects who achieved minimum (floor) or maximum (ceiling) scores. Note, for False alarm rate maximum score = 0% and minimum score = 100%.

**Emotional Recognition Task –Test-retest reliability**

| | Baseline (n = 49) | | Retest (n = 49) | | *Test-retest bias (%)* | *ICC* | *95% CI* |
|---|---|---|---|---|---|---|---|
| | *Mean* | *SD* | *Mean* | *SD* | | | |
| **Face version** | | | | | | | |
| False alarm rate (%) - Happy | 8.27 | 8.69 | 7.96 | 8.00 | -3.75 | 0.69 | 0.45–0.83 |
| False alarm rate (%) - Sad | 14.25 | 6.79 | 11.46 | 9.06 | -19.58 | 0.57 | 0.25–0.75 |
| False alarm rate (%) - Angry | 1.67 | 2.85 | 1.46 | 2.25 | -12.57 | 0.32 | -0.22–0.62 |
| False alarm rate (%) - Fearful | 3.33 | 3.32 | 2.59 | 3.79 | -22.22 | 0.66 | 0.40–0.81 |
| *d*-prime - Happy | 2.82 | 0.47 | 2.86 | 0.45 | 1.42 | 0.65 | 0.37–0.80 |
| *d*-prime - Sad | 2.28 | 0.57 | 2.48 | 0.54 | 8.77 | 0.59 | 0.29–0.77 |
| *d*-prime - Angry | 2.24 | 0.44 | 2.46 | 0.39 | 9.82 | 0.59 | 0.25–0.78 |
| *d*-prime - Fearful | 2.82 | 0.47 | 2.86 | 0.45 | 1.42 | 0.32 | -0.22–0.62 |
| **Eyes version** | | | | | | | |
| False alarm rate (%) - Happy | 13.81 | 8.80 | 12.21 | 9.22 | -11.59 | 0.62 | 0.33–0.79 |
| False alarm rate (%) - Sad | 13.50 | 7.03 | 12.18 | 8.15 | -9.78 | 0.65 | 0.38–0.80 |
| False alarm rate (%) - Angry | 3.50 | 3.82 | 3.27 | 3.86 | -6.57 | 0.45 | 0.01–0.69 |
| False alarm rate (%) - Fearful | 2.01 | 2.50 | 2.82 | 3.03 | 40.30 | 0.67 | 0.42–0.81 |
| *d*-prime - Happy | 2.71 | 0.49 | 2.69 | 0.45 | -0.74 | 0.45 | 0.03–0.69 |
| *d*-prime - Sad | 1.88 | 0.50 | 2.01 | 0.54 | 6.91 | 0.61 | 0.31–0.78 |
| *d*-prime - Angry | 2.31 | 0.40 | 2.49 | 0.46 | 7.79 | 0.48 | 0.11–0.70 |
| *d*-prime - Fearful | 2.71 | 0.49 | 2.69 | 0.45 | -0.74 | 0.43 | -0.02–0.68 |

**Legend:** Mean and SD are reported for baseline and retest sessions (3-5 weeks) along with Intraclass Correlation Coefficient (ICC) and their 95% Confidence Interval (95% CI). Test-retest bias is calculated as percentage change between first and second test: *Test-retest bias = ((retest-test)/test)*100.*

**Emotional Recognition Task – Correlations**

| | *Age* | *Sex*[$] | *Education* | *IQ* | *Neuroticism*[a] | *TMD* |
|---|---|---|---|---|---|---|
| **Face version** | | | | | | |
| False alarm rate (%) - Happy | 0.21 | 0.04 | 0.01 | -0.02 | -0.11 | -0.04 |
| False alarm rate (%) - Sad | 0.21 | -0.04 | 0.02 | -0.14 | 0.01 | -0.04 |
| False alarm rate (%) - Angry | 0.16 | -0.01 | -0.14 | **-0.26**** | -0.07 | -0.22 |
| False alarm rate (%) - Fearful | -0.17 | 0.01 | -0.06 | -0.13 | 0.09 | -0.08 |
| *d*-prime - Happy | **-0.26**** | -0.04 | -0.11 | 0.06 | 0.15 | 0.24 |
| *d*-prime - Sad | **-0.32**** | -0.12 | 0.05 | 0.21 | 0.17 | **0.28**** |
| *d*-prime - Angry | **-0.34***** | -0.09 | -0.09 | 0.23 | 0.11 | 0.04 |
| *d*-prime - Fearful | **-0.33***** | 0.04 | 0.03 | 0.19 | 0.09 | **0.26**** |
| **Eyes version** | | | | | | |
| False alarm rate (%) - Happy | 0.06 | -0.07 | 0.08 | 0.15 | -0.15 | 0.03 |
| False alarm rate (%) - Sad | -0.03 | 0.01 | 0.07 | -0.13 | 0.05 | -0.03 |
| False alarm rate (%) - Angry | 0.22 | -0.12 | 0.06 | -0.01 | 0.02 | -0.15 |
| False alarm rate (%) - Fearful | 0.04 | -0.16 | 0.05 | 0.09 | -0.17 | **-0.31**** |
| *d*-prime - Happy | -0.02 | 0.17 | -0.26 | -0.06 | 0.15 | 0.06 |
| *d*-prime - Sad | -0.17 | 0.17 | -0.11 | 0.07 | 0.23 | 0.24 |
| *d*-prime - Angry | -0.16 | 0.08 | -0.11 | 0.03 | 0.08 | 0.17 |
| *d*-prime - Fearful | **-0.29**** | 0.01 | -0.02 | 0.03 | 0.25 | 0.22 |

**Legend:** Correlations between Emotional Recognition Task (ERT) outcomes and age, sex, education indexed with the Family History Assessment Module (OS-FHAM) on a five-point likert scale, IQ score assessed with the Reynolds Intellectual Screening Test (RIST), total mood disturbance (TMD) indexed with the Profile of Mood Scale (POMS), and trait Neuroticism indexed with the NEO Personality Inventory-Revised (NEO PI-R, n = 93) and the NEO Personality Inventory 3 (NEO PI-3, n = 6). Correlation coefficients are reported as Spearman's rho; only $p$-values < .01 are considered significant and are marked in bold. ** = $p < .01$, *** = $p < .001$. [$] A negative rho value indicates males score higher while a positive rho value indicates females score higher. [a] N = 99 due to missing data from one participant.

## 2. Emotional Intensity Morphing Task

**Task description**

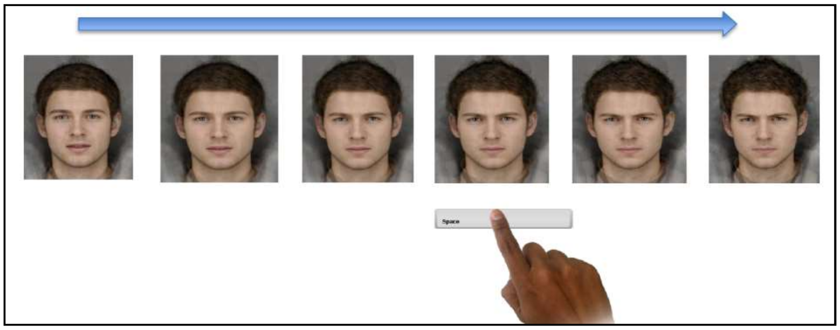

The Emotional Intensity Morphing task (IM) measures the perceptual threshold for the detection of emotions in facial expressions at different intensity levels. The task contains two conditions; an increase and a decrease condition. At the start of each trial, the participant is told which emotion to look for (happiness, sadness, anger, fear or disgust) before a face is shown whose emotional expression slowly morphs through 15 different intensity levels (1 = neutral, 15 = maximum intensity). For the increase condition, the emotional expression starts at neutral and morphs towards maximum intensity and the participant is instructed to press a button when they think they can see the emotion. For the decrease condition, the emotional expression starts at maximum intensity and morphs towards neutral and the participant is instructed to press a button when they think they can no longer see the emotion. For both conditions, each emotion is shown four times, equaling 40 trials in total.

**Main outcomes** (reported in main article)

- *Detection threshold for increase condition* – Average perceptual threshold for each emotion
- *Detection threshold for decrease condition* – Average perceptual threshold for each emotion

# 3. Face Affective Go/NoGo Task

**Task description**

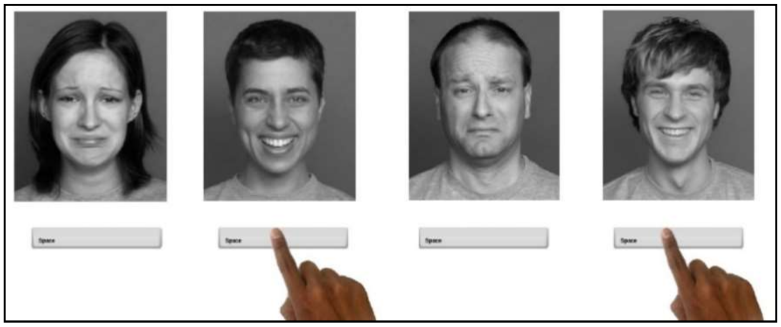

The Face Affective Go/NoGo Task (fAGN) measures attentional bias and behavioural inhibitory control. A series of emotional faces are shown and the participant is instructed to react to one specific type of emotion (e.g., happy faces) by pressing a button while refraining from reacting to another type of emotion (e.g., sad faces). The ratio of ‘Go’ to ‘NoGo’ stimuli is 50/50. The task contains six blocks: happy targets with neutral distractors (happy/neutral), happy targets with sad distractors (happy/sad), neutral targets with happy distractors (neutral/happy), neutral targets with sad distractors (neutral/sad), sad targets with happy distractors (sad/happy), and sad targets with neutral distractors (sad/neutral). Each block consists of 20 trials equaling 120 trials in total.

**Main outcomes** (reported in main article)

- *d-prime (d’)* – Index of discrimination sensitivity calculated as: *d’ = z(hits) - z(false alarms)*

**Secondary outcomes**[1]

- *Hits (H)* – Percentage of correct responses during Go-trials
- *False alarm (FA)* – Percentage of incorrect responses during NoGo-trials
- *Reaction time* – Reaction time (ms) for correct responses during Go-trials

[1]Note other possible secondary outcomes include Misses (i.e., failure to respond to Go-trials), and Correct rejections (i.e., correctly withholding response during NoGo-trials), but as these outcomes are the inverse of H and FA respectively and can be inferred by their counterparts they are not reported here.

**Face Affective Go/NoGo Task – Descriptive data**

| | *Mean* | *SD* | *Median* | *IQR* | *Range* | *Skewness* | *Floor effect* | *Ceiling effect* |
|---|---|---|---|---|---|---|---|---|
| Accuracy (%) - Happy/Neutral | *96.00* | *12.79* | 100.00 | 0.00 | 0–100 | -5.26 | 1% | 82% |
| Accuracy (%) - Happy/Sad | *95.20* | *13.14* | 100.00 | 2.50 | 0–100 | -4.99 | 1% | 75% |
| Accuracy (%) - Neutral/Happy | *86.60* | *20.16* | 90.00 | 20.00 | 10–100 | -1.95 | 0% | 48% |
| Accuracy (%) - Neutral/Sad | *80.70* | *22.17* | 90.00 | 30.00 | 10–100 | -1.35 | 0% | 33% |
| Accuracy (%) - Sad/Happy | *93.10* | *12.37* | 100.00 | 10.00 | 20–100 | -2.97 | 0% | 62% |
| Accuracy (%) - Sad/Neutral | *91.50* | *14.93* | 100.00 | 10.00 | 0–100 | -3.14 | 1% | 59% |
| False alarms (%) - Happy/Neutral | *7.80* | *9.91* | 0.00 | 10.00 | 0–40 | 5.26 | 0% | 51% |
| False alarms (%) - Happy/Sad | *9.30* | *10.47* | 10.00 | 10.00 | 0–40 | 4.99 | 0% | 43% |
| False alarms (%) - Neutral/Happy | *8.90* | *11.71* | 10.00 | 10.00 | 0–60 | 1.95 | 0% | 46% |
| False alarms (%) - Neutral/Sad | *12.70* | *13.40* | 10.00 | 20.00 | 0–50 | 1.35 | 0% | 34% |
| False alarms (%) - Sad/Happy | *9.30* | *10.47* | 10.00 | 10.00 | 0–50 | 2.97 | 0% | 41% |
| False alarms (%) - Sad/Neutral | *26.80* | *24.28* | 20.00 | 30.00 | 0–100 | 3.14 | 4% | 12% |
| Accuracy (ms) - Happy/Neutral | *420.30* | *56.52* | 406.52 | 76.20 | 307.29–619.02 | 0.88 | - | - |
| Accuracy (ms) - Happy/Sad | 438.30 | 56.99 | 435.56 | 72.37 | 296.12–577.24 | 0.28 | - | - |
| Accuracy (ms) - Neutral/Happy | 484.76 | 89.29 | 480.48 | 107.87 | 263.64–728.27 | 0.19 | - | - |
| Accuracy (ms) - Neutral/Sad | 526.40 | 89.41 | 516.30 | 110.12 | 297.8–736.81 | 0.13 | - | - |
| Accuracy (ms) - Sad/Happy | 476.95 | 71.51 | 480.80 | 113.40 | 306.61–639.37 | 0.12 | - | - |
| Accuracy (ms) - Sad/Neutral | 460.82 | 66.54 | 457.36 | 96.99 | 297.88–613.92 | 0.10 | - | - |

**Legend:** Mean, SD, median, interquartile range, range, and skewness are shown. Note, for outcomes with non-parametric data distribution, mean and SD are denoted in cursive gray indicating the median and interquartile range should be used as reference. Floor and ceiling effects are shown as percentage of test subjects who achieved minimum (floor) or maximum (ceiling) scores. Note, for False alarm rate maximum score = 0% and minimum score = 100%.

**Face Affective Go/NoGo Task – Test-retest reliability**

| | Baseline (n = 49) | | Retest (n = 49) | | *Test-retest bias (%)* | *ICC* | *95% CI* |
|---|---|---|---|---|---|---|---|
| | *Mean* | *SD* | *Mean* | *SD* | | | |
| Accuracy (%) - Happy/Neutral | 97.55 | 6.30 | 98.78 | 3.89 | 1.26 | 0.66 | 0.40–0.81 |
| Accuracy (%) - Happy/Sad | 94.08 | 16.82 | 97.96 | 6.12 | 4.12 | -0.02 | -0.78–0.42 |
| Accuracy (%) - Neutral/Happy | 86.33 | 19.33 | 92.45 | 16.40 | 7.09 | 0.26 | -0.27–0.58 |
| Accuracy (%) - Neutral/Sad | 78.57 | 22.27 | 85.31 | 18.27 | 8.58 | 0.02 | -0.70–0.44 |
| Accuracy (%) - Sad/Happy | 93.47 | 10.52 | 94.90 | 9.60 | 1.53 | 0.30 | -0.24–0.61 |
| Accuracy (%) - Sad/Neutral | 92.86 | 10.61 | 96.33 | 7.27 | 3.74 | 0.33 | -0.15–0.61 |
| False alarms (%) - Happy/Neutral | 6.73 | 8.75 | 5.71 | 7.36 | -15.16 | 0.33 | -0.19–0.62 |
| False alarms (%) - Happy/Sad | 7.96 | 10.99 | 8.78 | 8.81 | 10.30 | 0.22 | -0.40–0.56 |
| False alarms (%) - Neutral/Happy | 8.98 | 9.84 | 5.92 | 8.14 | -34.08 | 0.56 | 0.24–0.75 |
| False alarms (%) - Neutral/Sad | 14.69 | 14.16 | 11.84 | 13.95 | -19.40 | 0.39 | -0.08–0.65 |
| False alarms (%) - Sad/Happy | 6.73 | 7.47 | 9.80 | 11.08 | 45.62 | 0.33 | -0.16–0.61 |
| False alarms (%) - Sad/Neutral | 24.90 | 23.99 | 20.00 | 21.21 | -19.68 | 0.45 | 0.03–0.69 |
| Accuracy (ms) - Happy/Neutral | 413.82 | 54.45 | 423.20 | 56.72 | 2.27 | 0.59 | 0.27–0.77 |
| Accuracy (ms) - Happy/Sad | 437.27 | 61.64 | 442.57 | 61.80 | 1.21 | 0.63 | 0.34–0.79 |
| Accuracy (ms) - Neutral/Happy | 490.24 | 97.54 | 491.45 | 75.22 | 0.25 | 0.68 | 0.42–0.82 |
| Accuracy (ms) - Neutral/Sad | 533.64 | 82.67 | 538.30 | 87.19 | 0.87 | 0.56 | 0.22–0.75 |
| Accuracy (ms) - Sad/Happy | 487.26 | 68.28 | 492.43 | 66.59 | 1.06 | 0.48 | 0.08–0.71 |
| Accuracy (ms) - Sad/Neutral | 465.23 | 62.04 | 481.79 | 70.20 | 3.56 | 0.59 | 0.28–0.77 |

**Legend:** Mean and SD are reported for baseline and retest sessions (3-5 weeks) along with Intraclass Correlation Coefficient (ICC) and their 95% Confidence Interval (95% CI). Test-retest bias is calculated as percentage change between first and second test: *Test-retest bias = ((retest-test)/test)*100.*

**Face Affective Go/NoGo Task – Correlations**

| | *Age* | *Sex*[$] | *Education* | *IQ* | *Neuroticism*[a] | *TMD* |
|---|---|---|---|---|---|---|
| Accuracy (%) - Happy/Neutral | -0.15 | 0.13 | -0.07 | -0.02 | -0.02 | -0.04 |
| Accuracy (%) - Happy/Sad | -0.07 | 0.03 | 0.02 | 0.02 | 0.02 | -0.03 |
| Accuracy (%) - Neutral/Happy | -0.01 | 0.004 | 0.02 | -0.17 | 0.06 | 0.01 |
| Accuracy (%) - Neutral/Sad | -0.17 | -0.03 | 0.12 | 0.13 | 0.03 | 0.24 |
| Accuracy (%) - Sad/Happy | -0.08 | -0.06 | 0.09 | 0.003 | 0.11 | 0.08 |
| Accuracy (%) - Sad/Neutral | -0.14 | 0.18 | 0.01 | 0.00 | 0.08 | 0.24 |
| False alarms (%) - Happy/Neutral | -0.08 | -0.08 | -0.05 | -0.08 | 0.03 | 0.03 |
| False alarms (%) - Happy/Sad | -0.10 | -0.13 | 0.08 | -0.03 | -0.03 | -0.05 |
| False alarms (%) - Neutral/Happy | -0.04 | 0.06 | -0.11 | -0.18 | 0.01 | 0.05 |
| False alarms (%) - Neutral/Sad | **-0.27**** | 0.13 | -0.18 | 0.02 | -0.10 | -0.10 |
| False alarms (%) - Sad/Happy | -0.09 | 0.13 | -0.02 | -0.09 | 0.15 | 0.08 |
| False alarms (%) - Sad/Neutral | 0.03 | -0.16 | 0.17 | -0.01 | 0.07 | -0.01 |
| Accuracy (ms) - Happy/Neutral | 0.13 | -0.01 | -0.03 | -0.18 | 0.02 | -0.02 |
| Accuracy (ms) - Happy/Sad | 0.21 | -0.04 | -0.02 | -0.20 | 0.10 | -0.02 |
| Accuracy (ms) - Neutral/Happy | 0.15 | -0.053 | -0.01 | 0.03 | 0.05 | -0.11 |
| Accuracy (ms) - Neutral/Sad | 0.22 | -0.04 | 0.20 | 0.14 | 0.05 | -0.03 |
| Accuracy (ms) - Sad/Happy | 0.13 | -0.11 | -0.08 | 0.203 | 0.06 | -0.06 |
| Accuracy (ms) - Sad/Neutral | **0.29**** | -0.03 | -0.05 | 0.06 | -0.05 | -0.02 |

**Legend:** Correlations between Face Affective Go/NoGo task (fAGN) outcomes and age, sex, education indexed with the Family History Assessment Module (OS-FHAM) on a five-point likert scale, IQ score assessed with the Reynolds Intellectual Screening Test (RIST), total mood disturbance (TMD) indexed with the Profile of Mood Scale (POMS), and trait Neuroticism indexed with the NEO Personality Inventory-Revised (NEO PI-R, n = 93) and the NEO Personality Inventory 3 (NEO PI-3, n = 6). Correlation coefficients are reported as Spearman's rho; only $p$-values < .01 are considered significant and are marked in bold. ** = $p$ < .01. [$] A negative rho value indicates males score higher while a positive rho value indicates females score higher. [a] N = 99 due to missing data from one participant.

# 4. Reinforcement Learning Task

**Task description**

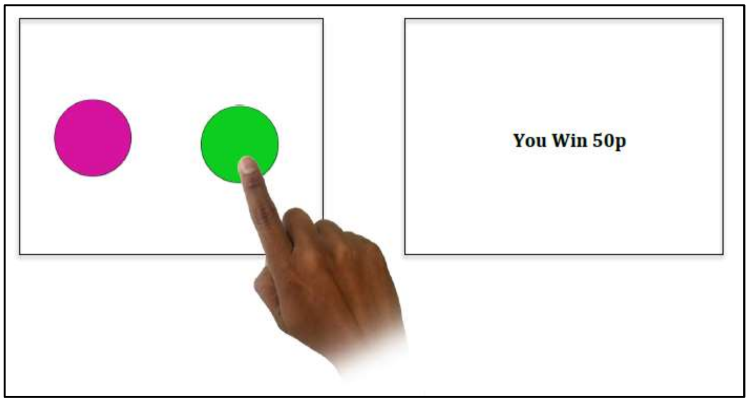


The Reinforcement Learning Task assesses learning based on reward and punishment feedback. In every trial, two colored circles appear and the participant is asked to make a choice between the two. Within each color pair, the ratio between favorable and unfavorable outcomes for one color is 70/30 while the ratio between favorable and unfavorable outcome for the other color is 30/70. In total, there are four pairs of colors (grey/red, yellow/black, orange/blue, and purple/green). Half the color-pairs represent a win condition in which the favorable outcome is a monetary reward while the unfavorable outcome is no reward and the other half of the color-pairs represent a loss condition in which the favorable outcome is no loss and the unfavorable outcome is monetary loss. The task contains a learning phase made up of 120 trials and a transfer phase in which the colors are paired randomly made up of 48 trials, equaling a total of 168 trials.

**Main outcomes** (reported in main article)

- *Learning rate alpha for win and loss conditions*– Index of learning speed in the context of positive and negative feedback calculated using a reinforcement learning algorithm

**Secondary outcomes**

- *Temperature for win and loss conditions* – Index of exploration vs static behavior calculated using a reinforcement learning algorithm

**Reinforcement Learning Task – Descriptive data**

| | *Mean* | *SD* | *Median* | *IQR* | *Range* | *Skewness* | *Floor effect* | *Ceiling effect* |
|---|---|---|---|---|---|---|---|---|
| Temperature - Win condition | *0.75* | *1.27* | 0.05 | 0.82 | 0.01–4.58 | 1.84 | 32% | 0% |
| Temperature - Loss condition | *0.92* | *0.99* | 0.61 | 1.05 | 0.01–4.17 | 1.56 | 32% | 0% |

**Legend:** N = 68, as 32 participants performed below chance level, violating the assumptions of the reinforcement learning algorithm used to determine the temperature outcome. Mean, SD, median, interquartile range, range, and skewness are shown. Note, for outcomes with non-parametric data distribution, mean and SD are denoted in cursive gray indicating the median and interquartile range should be used as reference. Floor and ceiling effects are shown as percentage of test subjects who achieved minimum (floor) or maximum (ceiling) scores. Note, floor and ceiling effects are here based on the percentage of trials in which the correct (i.e., most favorable) colored circle is chosen; this data is not shown but should be inspected prior to running the reinforcement learning algorithm. Thus, to reach the criteria for floor effect the most favorable colored circled must be chosen in 50% > of trials (indicating a hit rate of below chance level).

**Reinforcement Learning Task – Test-retest reliability**

| | **Baseline (n = 35)** | | **Retest (n = 35)** | | *Test-retest bias (%)* | *ICC* | *95% CI* |
|---|---|---|---|---|---|---|---|
| | *Mean* | *SD* | *Mean* | *SD* | | | |
| Temperature - Win condition | 0.91 | 1.38 | 0.80 | 1.27 | -11.87 | 0.18 | -0.48–0.54 |
| Temperature - Loss condition | 0.97 | 1.02 | 1.13 | 1.46 | 17.09 | -0.07 | -0.94–0.41 |

**Legend:** N = 35, as 14 participants performed below chance level, violating the assumptions of the reinforcement learning algorithm used to determine the temperature outcome. Mean and SD are reported for baseline and retest sessions (3-5 weeks) along with Intraclass Correlation Coefficient (ICC) and their 95% Confidence Interval (95% CI). Test-retest bias is calculated as percentage change between first and second test: *Test-retest bias = ((retest-test)/test)*100.*

**Reinforcement Learning Task – Correlations**

| | *Age* | *Sex*[$] | *Education* | *IQ* | *Neuroticism*[a] | *TMD* | *Motivation* | *Diligence* |
|---|---|---|---|---|---|---|---|---|
| Temperature - Win condition | -0.01 | 0.04 | 0.08 | 0.09 | -0.09 | -0.19 | **-0.34**** | **-0.35**** |
| Temperature - Loss condition | 0.12 | -0.11 | -0.12 | 0.07 | -0.17 | -0.16 | -0.24 | **-0.31**** |

**Legend:** N = 68, as 32 participants performed below chance level, violating the assumptions of the reinforcement learning algorithm used to determine the temperature outcome. Correlations between Reinforcement Learning outcomes and age, sex, education indexed with the Family History Assessment Module (OS-FHAM) on a five-point likert scale, IQ score assessed with the Reynolds Intellectual Screening Test (RIST), total mood disturbance (TMD) indexed with the Profile of Mood Scale (POMS), and trait Neuroticism indexed with the NEO Personality Inventory-Revised (NEO PI-R, n = 62) and the NEO Personality Inventory 3 (NEO PI-3, n = 5). Correlations between self-reported motivation and diligence are also shown. Correlation coefficients are reported as Spearman's rho; only *p*-values < .01 are considered significant and are marked in bold. ** = $p < .01$. [$] A negative rho value indicates males score higher while a positive rho value indicates females score higher. [a] N = 67 due to missing data from one participant.

# 5. Monetary Incentive Reward Task

**Task description**

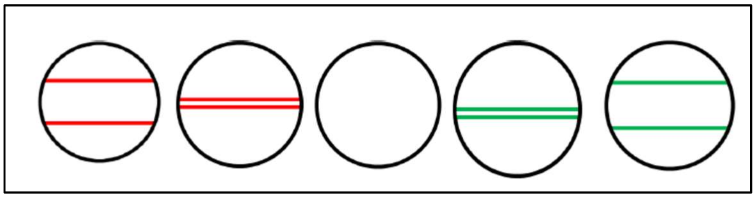

The Monetary Incentive Reward task (MIR) assesses sensitivity to reward and punishment. Each trial, a pair of circles appear on the screen followed by a black box. Participants are instructed to press a key as soon as they see the black box appear. The difficulty level of the task is tailored to the individual reaction time of each participant, calculated from 30 baseline trials. In the main task, two lines appear in the circles indicating the potential to win money (green lines) or lose money (red lines). The distance between the lines indicate the size of the gain/loss, with large distances signaling a large gain/loss and small distances signaling a small gain/loss. Participants must respond faster than their baseline reaction time in order to gain money or avoid loss and are given feedback after each individual trial. Each condition (high win, low win, high loss, low loss, and neutral) are shown 20 times, equaling 100 trials in total as well as 30 neutral baseline trials.

**Main outcomes** (reported in the main article)

- *Reaction time (ms) for win* – Average reaction time in milliseconds across the two win conditions relative to baseline calculated as: *Win = baseline - (high win + low win)/2*
- *Reaction time (ms) for loss* – Average reaction time in milliseconds across the two loss conditions relative to baseline calculated as: *Loss = baseline - (high loss + low loss)/2*

**Secondary outcomes**

- *Reaction time for each condition* – Average reaction time in milliseconds for high win, low win, high loss, low loss relative to baseline, e.g., calculated as: *High win = baseline - high win*

**Monetary Incentive Reward Task – Descriptive data**

| | *Mean* | *SD* | *Median* | *IQR* | *Range* | *Skewness* | *Floor effect* | *Ceiling effect* |
|---|---|---|---|---|---|---|---|---|
| Reaction time (ms) - High win | 17.02 | 20.31 | 17.96 | 27.39 | -26.53–78.93 | 0.41 | - | - |
| Reaction time (ms) - Low win | 17.79 | 20.33 | 17.59 | 26.00 | -34.04–67.5 | 0.22 | - | - |
| Reaction time (ms) - High loss | 19.60 | 21.15 | 18.67 | 20.32 | -38.95–94.53 | 0.26 | - | - |
| Reaction time (ms) - Low loss | *17.87* | *18.34* | 15.90 | 23.65 | -50.08–74.76 | 0.19 | - | - |

**Legend:** Mean, SD, median, interquartile range, range, and skewness are shown. Note, for outcomes with non-parametric data distribution, mean and SD are denoted in cursive gray indicating the median and interquartile range should be used as reference. Note floor and ceiling effects cannot be estimated for reaction time outcomes.

**Monetary Incentive Reward Task – Test-retest reliability**

| | **Baseline (n = 49)** | | **Retest (n = 49)** | | *Test-retest bias (%)* | *ICC* | *95% CI* |
|---|---|---|---|---|---|---|---|
| | *Mean* | *SD* | *Mean* | *SD* | | | |
| Reaction time (ms) - High win | 17.10 | 22.16 | 20.76 | 25.58 | 21.40 | -0.64 | -1.96–0.08 |
| Reaction time (ms) - Low win | 19.85 | 20.15 | 13.12 | 19.64 | -33.90 | 0.02 | -0.68–0.44 |
| Reaction time (ms) - High loss | 21.01 | 23.50 | 22.12 | 24.47 | 5.28 | -0.63 | -1.96–0.10 |
| Reaction time (ms) - Low loss | 17.10 | 21.17 | 15.85 | 20.10 | -7.31 | 0.03 | -0.75–0.46 |

**Legend:** Mean and SD are reported for baseline and retest sessions (3-5 weeks) along with Intraclass Correlation Coefficient (ICC) and their 95% Confidence Interval (95% CI). Test-retest bias is calculated as percentage change between first and second test: *Test-retest bias = ((retest-test)/test)*100.*

**Monetary Incentive Reward Task – Correlations**

| | *Age* | *Sex*[$] | *Education* | *IQ* | *Neuroticism*[a] | *TMD* | *Motivation* | *Diligence* |
|---|---|---|---|---|---|---|---|---|
| Reaction time (ms) - High win | -0.03 | -0.01 | 0.01 | -0.12 | 0.02 | -0.14 | -0.08 | 0.09 |
| Reaction time (ms) - Low win | -0.10 | 0.00 | 0.22 | -0.10 | -0.11 | -0.19 | -0.09 | 0.08 |
| Reaction time (ms) - High loss | 0.01 | 0.07 | -0.04 | -0.12 | -0.07 | -0.19 | -0.14 | 0.06 |
| Reaction time (ms) - Low loss | 0.02 | 0.05 | -0.09 | -0.09 | -0.04 | -0.11 | 0.01 | 0.09 |

**Legend:** Correlations between Monetary Incentive Reward Task outcomes and age, sex, education indexed with the Family History Assessment Module (OS-FHAM) on a five-point likert scale, IQ score assessed with the Reynolds Intellectual Screening Test (RIST), total mood disturbance (TMD) indexed with the Profile of Mood Scale (POMS), and trait Neuroticism indexed with the NEO Personality Inventory-Revised (NEO PI-R, n = 93) and the NEO Personality Inventory 3 (NEO PI-3, n = 6). Correlations between self-reported motivation and diligence are also shown. Correlation coefficients are reported as Spearman's rho; only *p*-values < .01 are considered significant and are marked in bold. [$] A negative rho value indicates males score higher while a positive rho value indicates females score higher. [a] N = 99 due to missing data from one participant.

# 6. Progressive Ratio Task

**Task description**

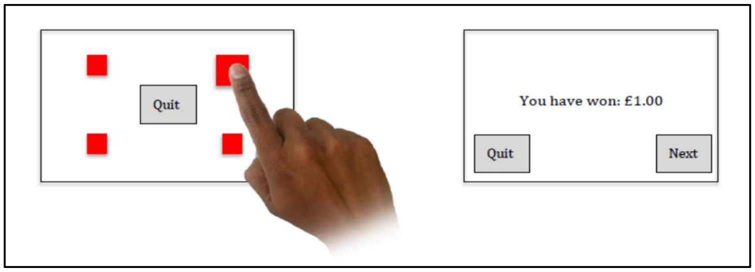


The Progressive Ratio Task assesses self-control and motivational breakpoint. Four squares are shown on a screen and the participant is instructed to pick the odd one out. The task is made up of a high-yield block (reward 10 Danish kroners), a medium-yield block (reward 2 Danish kroners), and a low-yield block (reward ½ Danish kroners). Within each block, the number of trials needed to obtain a monetary reward continually doubles (4, 8, 16, 32, etc.). The participant is told that they can quit the task at any time, however they must remain in front of the computer until the full run-time of the task is complete (~20 minutes). The high-yield block contains 60 trials, the medium-yield block contains 124 trials, and the low-yield block contains 252 trials, equaling 436 trials in total.

**Main outcomes** (reported in the main article)

- *Breakpoint* – Number of trials completed

**Secondary outcomes**

- *Post reinforcement break* – Average time in seconds between reward and decision to continue task calculated for each block

**Progressive Ratio Task – Descriptive data**

| | *N* | *Mean* | *SD* | *Median* | *IQR* | *Range* | *Skewness* | *Floor effect* | *Ceiling effect* |
|---|---|---|---|---|---|---|---|---|---|
| Post reinforcement pause (s) – High-yield | 98 | *1.11* | *1.07* | 0.79 | 0.63 | 0.28–8.66 | 4.53 | - | - |
| Post reinforcement pause (s) – Medium-yield | 90 | *0.68* | *0.69* | 0.49 | 0.29 | 0.16–4.85 | 4.32 | - | - |
| Post reinforcement pause (s) - Low-yield | 73 | *0.55* | *0.42* | 0.44 | 0.34 | 0.15–2.77 | 3.00 | - | - |

**Legend:** N indicates number of participants who completed each block. Mean, SD, median, interquartile range, range, and skewness are shown. Note, for outcomes with non-parametric data distribution, mean and SD are denoted in cursive gray indicating the median and interquartile range should be used as reference. Note floor and ceiling effects cannot be estimated for outcomes measured in time.

**Progressive Ratio Task – Test-retest reliability**

| | **Baseline (n = 49)** | | | **Retest (n = 49)** | | | *Test-retest bias (%)* | *ICC* | *95% CI* |
|---|---|---|---|---|---|---|---|---|---|
| | *N* | *Mean* | *SD* | *N* | *Mean* | *SD* | | | |
| Post reinforcement pause (s) – High-yield | 48 | 1.14 | 0.71 | 49 | 0.63 | 0.23 | -44.74 | 0.26 | -0.16–0.56 |
| Post reinforcement pause (s) - Medium-yield | 43 | 0.78 | 0.71 | 48 | 0.69 | 1.72 | -11.54 | 0.09 | -0.71–0.51 |
| Post reinforcement pause (s) - Low-yield | 35 | 0.68 | 0.56 | 38 | 0.67 | 1.01 | -1.47 | -0.13 | -1.56–0.49 |

**Legend:** N indicates number of participants who completed each block. Mean and SD are reported for baseline and retest sessions (3-5 weeks) along with Intraclass Correlation Coefficient (ICC) and their 95% Confidence Interval (95% CI). Test-retest bias is calculated as percentage change between first and second test: *Test-retest bias = ((retest-test)/test)*100.*

**Progressive Ratio Task – correlations**

| | *N* | *Age* | *Sex*[$] | *Education* | *IQ* | *Neuroticism*[a] | *TMD* | *Motivation* | *Diligence* |
|---|---|---|---|---|---|---|---|---|---|
| Post reinforcement pause (s) - High-yield | 98 | 0.09 | 0.10 | 0.09 | -0.12 | -0.06 | 0.03 | -0.02 | 0.03 |
| Post reinforcement pause (s) - Medium-yield | 90 | 0.07 | 0.12 | -0.03 | -0.09 | 0.07 | 0.06 | 0.05 | 0.06 |
| Post reinforcement pause (s) - Low-yield | 73 | 0.11 | 0.16 | 0.01 | 0.02 | 0.12 | 0.05 | -0.03 | 0.10 |

**Legend:** N indicates number of participants who completed each block. Correlations between Progressive Ratio Task outcomes and age, sex, education indexed with the Family History Assessment Module (OS-FHAM) on a five-point likert scale, IQ score assessed with the Reynolds Intellectual Screening Test (RIST), total mood disturbance (TMD) indexed with the Profile of Mood Scale (POMS), and trait Neuroticism indexed with the NEO Personality Inventory-Revised (NEO PI-R, n = 93) and the NEO Personality Inventory 3 (NEO PI-3, n = 6). Correlations between self-reported motivation and diligence are also shown. Correlation coefficients are reported as Spearman's rho; only *p*-values < .01 are considered significant and are marked in bold. [$] A negative rho value indicates males score higher while a positive rho value indicates females score higher. [a] N = 99 due to missing data from one participant.

# 7. Adapted Cambridge Gambling Task

## Task description

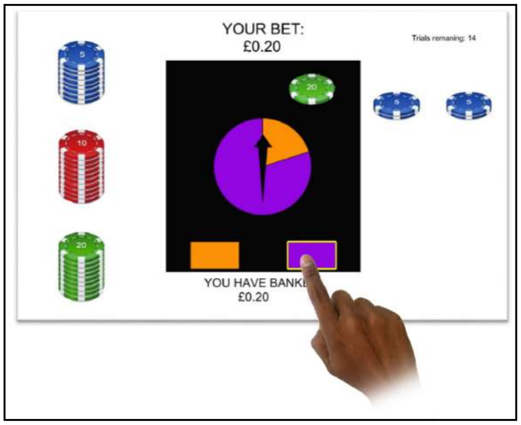


The Adapted Cambridge Gambling Task (aCGT) assesses risk-taking and decision-making. At the beginning of the task, the participant is given three stacks of chips; 10 chips worth 1.5 Danish kroners, 10 chips worth 2 Danish kroners, and 10 chips worth 4 Danish kroners. In each trial, a roulette wheel made up of two colors is presented; the participant must pick a color and place two chips on their bet. The task contains two conditions; a win and a loss condition. In the win condition, the participant will either double or keep the money they bet and in the loss condition, the participant will either keep or lose the money they bet. Each condition contains 15 trials (2 x 90% odds, 2 x 80% odds, 4 x 70% odds, 4 x 60% odds, and 3 x 50% odds), equaling 30 trials in total.

**Main outcomes** (reported in the main article)

- *Risk adjustment for win and loss conditions* – Index of association between odds of winning and the size of bet calculated as: *Risk adjustment = ((2 x mean bet 90%) + (1 x mean bet 80%) + (0 x mean bet 70%) - (1 x mean bet 60%) - (2 x mean bet 50%)) / average bet*.

**Secondary outcomes**

- *Mean bets for win and loss conditions* – Mean bet in kroners for individual odds (90%, 80%, 70%, 60%, and 50%) for both win and loss conditions.
- *Quality of decision making* – Percentage of trials in which the best odds were picked

**Adapted Cambridge Gambling Task – Descriptive data**

| | *Mean* | *SD* | *Median* | *IQR* | *Range* | *Skewness* | *Floor effect* | *Ceiling effect* |
|---|---|---|---|---|---|---|---|---|
| Mean bet 50% (kroners) - Win condition | *1.57* | *0.54* | 1.42 | 1.00 | 1–3 | 0.72 | - | - |
| Mean bet 60% (kroners) - Win condition | *2.04* | *0.54* | 2.00 | 0.69 | 1–3.50 | 0.49 | - | - |
| Mean bet 70% (kroners) - Win condition | 2.54 | 0.51 | 2.50 | 0.62 | 1.38–4 | -0.07 | - | - |
| Mean bet 80% (kroners) - Win condition | *2.90* | *0.77* | 3.00 | 1.00 | 1–4 | -0.31 | - | - |
| Mean bet 90% (kroners) - Win condition | *3.15* | *0.85* | 3.00 | 1.50 | 1.25–4 | -0.57 | - | - |
| Mean bet 50% (kroners) - Loss condition | *1.47* | *0.50* | 1.33 | 0.71 | 1–3.17 | 1.13 | - | - |
| Mean bet 60% (kroners) - Loss condition | *1.81* | *0.45* | 1.75 | 0.50 | 1–3.50 | 1.13 | - | - |
| Mean bet 70% (kroners) - Loss condition | *2.58* | *0.49* | 2.56 | 0.53 | 1.33–4 | 0.36 | - | - |
| Mean bet 80% (kroners) - Loss condition | *3.21* | *0.72* | 3.00 | 1.25 | 1–4 | -0.68 | - | - |
| Mean bet 90% (kroners) - Loss condition | *3.37* | *0.69* | 3.50 | 1.00 | 1.50–4 | -0.79 | - | - |
| Quality of decision making | *97.53* | *5.62* | 100.00 | 3.33 | 60–100 | -4.26 | 0% | 66% |

**Legend:** Mean, SD, median, interquartile range, range, and skewness are shown. Note, for outcomes with non-parametric data distribution, mean and SD are denoted in cursive gray indicating the median and interquartile range should be used as reference. Floor and ceiling effects are shown as percentage of test subjects who achieved minimum (floor) or maximum (ceiling) scores. Note, floor and ceiling effects cannot be estimated for monetary bets.

**Adapted Cambridge Gambling Task – Test-retest reliability**

| | Baseline (n = 49) | | Retest (n = 49) | | *Test-retest bias (%)* | *ICC* | *95% CI* |
|---|---|---|---|---|---|---|---|
| | *Mean* | *SD* | *Mean* | *SD* | | | |
| Mean bet 50% (kroners) - Win condition | 1.59 | 0.54 | 1.41 | 0.44 | -11.32 | 0.44 | 0.04–0.68 |
| Mean bet 60% (kroners) - Win condition | 1.96 | 0.53 | 1.84 | 0.40 | -6.12 | 0.28 | -0.26–0.59 |
| Mean bet 70% (kroners) - Win condition | 2.55 | 0.55 | 2.52 | 0.51 | -1.18 | 0.36 | -0.16–0.64 |
| Mean bet 80% (kroners) - Win condition | 2.98 | 0.78 | 3.16 | 0.71 | 6.04 | -0.02 | -0.81–0.43 |
| Mean bet 90% (kroners) - Win condition | 3.17 | 0.87 | 3.51 | 0.70 | 10.73 | 0.38 | -0.05–0.65 |
| Mean bet 50% (kroners) - Loss condition | 1.46 | 0.50 | 1.39 | 0.39 | -4.79 | -0.06 | -0.90–0.40 |
| Mean bet 60% (kroners) - Loss condition | 1.83 | 0.50 | 1.76 | 0.49 | -3.83 | 0.48 | 0.08–0.71 |
| Mean bet 70% (kroners) - Loss condition | 2.56 | 0.43 | 2.49 | 0.44 | -2.73 | 0.37 | -0.11–0.65 |
| Mean bet 80% (kroners) - Loss condition | 3.26 | 0.72 | 3.44 | 0.66 | 5.52 | 0.09 | -0.59–0.48 |
| Mean bet 90% (kroners) - Loss condition | 3.37 | 0.69 | 3.52 | 0.69 | 4.45 | 0.10 | -0.58–0.49 |
| Quality of decision making | 97.96 | 6.04 | 99.18 | 1.45 | 1.25 | 0.22 | -0.36–0.56 |

**Legend:** Mean and SD are reported for baseline and retest sessions (3-5 weeks) along with Intraclass Correlation Coefficient (ICC) and their 95% Confidence Interval (95% CI). Test-retest bias is calculated as percentage change between first and second test: *Test-retest bias = ((retest-test)/test)*100.*

**Adapted Cambridge Gambling Task – Correlations**

| | *Age* | *Sex*[$] | *Education* | *IQ* | *Neuroticism*[a] | *TMD* | *Motivation* | *Diligence* |
|---|---|---|---|---|---|---|---|---|
| Mean bet 50% (kroners) - Win condition | -0.11 | 0.16 | -0.05 | -0.05 | 0.16 | 0.09 | 0.03 | 0.03 |
| Mean bet 60% (kroners) - Win condition | 0.03 | 0.20 | -0.03 | -0.25 | 0.06 | -0.08 | 0.15 | 0.09 |
| Mean bet 70% (kroners) - Win condition | -0.09 | -0.01 | -0.01 | -0.04 | -0.03 | -0.09 | 0.06 | 0.04 |
| Mean bet 80% (kroners) - Win condition | 0.18 | -0.26 | -0.12 | 0.11 | -0.04 | 0.03 | 0.08 | 0.07 |
| Mean bet 90% (kroners) - Win condition | 0.00 | -0.16 | 0.22 | 0.18 | -0.17 | 0.07 | -0.17 | -0.06 |
| Mean bet 50% (kroners) - Loss condition | 0.16 | 0.16 | 0.00 | -0.06 | 0.12 | 0.03 | 0.00 | -0.05 |
| Mean bet 60% (kroners) - Loss condition | 0.15 | -0.07 | -0.14 | -0.02 | -0.04 | -0.08 | -0.09 | -0.09 |
| Mean bet 70% (kroners) - Loss condition | -0.03 | -0.09 | -0.06 | -0.15 | 0.12 | 0.04 | 0.03 | 0.05 |
| Mean bet 80% (kroners) - Loss condition | -0.20 | -0.11 | 0.16 | 0.12 | -0.22 | -0.02 | 0.02 | 0.03 |
| Mean bet 90% (kroners) - Loss condition | -0.09 | 0.15 | 0.19 | 0.12 | -0.06 | 0.00 | 0.03 | 0.05 |
| Quality of decision making | -0.05 | 0.32 | 0.01 | 0.19 | -0.02 | -0.01 | -0.18 | -0.04 |

**Legend:** Correlations between Adapted Cambridge Gambling Task outcomes and age, sex, education indexed with the Family History Assessment Module (OS-FHAM) on a five-point likert scale, IQ score assessed with the Reynolds Intellectual Screening Test (RIST), total mood disturbance (TMD) indexed with the Profile of Mood Scale (POMS), and trait Neuroticism indexed with the NEO Personality Inventory-Revised (NEO PI-R, n = 93) and the NEO Personality Inventory 3 (NEO PI-3, n = 6). Correlations between self-reported motivation and diligence are also shown. Correlation coefficients are reported as Spearman's rho; only *p*-values < .01 are considered significant and are marked in bold. [$] A negative rho value indicates males score higher while a positive rho value indicates females score higher. [a] N = 99 due to missing data from one participant.

# 8. Moral Emotions Task

## Task description

The Moral Emotions Task (ME) assesses the effect of intention on experience of moral emotions.

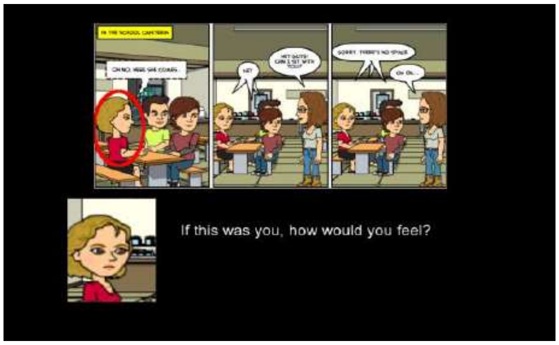


A series of moral scenarios are shown in which one character either intentionally or unintentionally causes another character physical or emotional harm. The participant is instructed to imagine themselves as either the agent (i.e., the person causing the harm) or the victim and to rate on a scale from 1 (not at all) to 7 (extremely) how ‘guilty’, ‘ashamed’, and ‘annoyed’ they would feel as well as how overall ‘bad’ to ‘good’ they would feel. The task consists of 12 moral scenarios and each scenario is shown twice so the participant can take on the role of both agent and victim, equaling a total of 24 trials.

**Main outcomes** (reported in the main article)

- *Agent and victim ratings of guilt and shame* – Average ratings of guilt and shame for agent and victim scenarios e.g., calculated as: *Agent guilt = (agent guilt intentional + agent guilt unintentional)/2*.

## Secondary outcomes

- *Ratings for each condition (role x intentionality x emotion)* – Ratings for each of the 16 possible combinations of agent vs victim, intentional vs unintentional, and ‘guilt’, ‘shame’, ‘annoyance’[2], and ‘feeling bad’.

[2] As discussed in the main article, we observed a serious issue with the ratings for ‘annoyance’. As a consequence, this outcome will not be presented here.

**Moral Emotions Task – Descriptive data**

| | *Mean* | *SD* | *Median* | *IQR* | *Range* | *Skewness* | *Floor effect* | *Ceiling effect* |
|---|---|---|---|---|---|---|---|---|
| Guilt - Agent intentional | *6.14* | *0.85* | 6.33 | 0.83 | 2.50–7.00 | -1.88 | 0% | 13% |
| Guilt - Agent unintentional | *5.58* | *0.82* | 5.71 | 0.86 | 2.00–7.00 | -1.61 | 0% | 1% |
| Guilt - Victim intentional | *1.63* | *0.63* | 1.50 | 0.83 | 1.00–3.83 | 1.35 | 19% | 0% |
| Guilt - Victim unintentional | *1.54* | *0.59* | 1.43 | 0.86 | 1.00–4.43 | 1.88 | 26% | 0% |
| Shame - Agent intentional | *5.99* | *0.87* | 6.17 | 1.04 | 2.50–7.00 | -1.46 | 0% | 10% |
| Shame - Agent unintentional | *5.48* | *0.88* | 5.57 | 1.14 | 2.00–7.00 | -1.12 | 0% | 1% |
| Shame - Victim intentional | *2.46* | *0.96* | 2.25 | 1.67 | 1.00–4.83 | 0.26 | 9% | 0% |
| Shame - Victim unintentional | *1.47* | *0.63* | 1.14 | 0.75 | 1.00–4.00 | 1.57 | 38% | 0% |
| Bad - Agent intentional | *1.92* | *0.70* | 1.83 | 1.00 | 1.00–4.83 | 1.11 | 0% | 8% |
| Bad - Agent unintentional | *2.30* | *0.72* | 2.29 | 0.75 | 1.00–5.57 | 1.38 | 0% | 3% |
| Bad - Victim intentional | *2.54* | *0.69* | 2.50 | 0.67 | 1.00–5.17 | 1.17 | 0% | 1% |
| Bad - Victim unintentional | *3.06* | *0.61* | 3.00 | 0.75 | 1.57–5.43 | 0.65 | 0% | 0% |

**Legend:** Mean, SD, median, interquartile range, range, and skewness are shown. Note, for outcomes with non-parametric data distribution, mean and SD are denoted in cursive gray indicating the median and interquartile range should be used as reference. Floor and ceiling effects are shown as percentage of test subjects who achieved minimum (floor) or maximum (ceiling) scores. Note, ratings for bad are reversed so a score of 1 is high (i.e., feeling ‘bad’) and a score of 7 is low (i.e., feeling ‘good’).

**Moral Emotions Task – Test-retest reliability**

| | **Baseline (n = 49)** | | **Retest (n = 49)** | | *Test-retest bias (%)* | *ICC* | *95% CI* |
|---|---|---|---|---|---|---|---|
| | *Mean* | *SD* | *Mean* | *SD* | | | |
| Guilt - Agent intentional | 6.18 | 0.83 | 6.12 | 0.69 | -0.97 | 0.73 | 0.51–0.85 |
| Guilt - Agent unintentional | 5.59 | 0.86 | 5.59 | 0.84 | 0 | 0.85 | 0.74–0.92 |
| Guilt - Victim intentional | 1.69 | 0.67 | 1.71 | 0.57 | 1.18 | 0.75 | 0.56–0.86 |
| Guilt - Victim unintentional | 1.57 | 0.55 | 1.66 | 0.55 | 5.73 | 0.80 | 0.65–0.89 |
| Shame - Agent intentional | 6.06 | 0.86 | 5.92 | 0.78 | -2.31 | 0.80 | 0.65–0.89 |
| Shame - Agent unintentional | 5.54 | 0.92 | 5.44 | 0.82 | -1.81 | 0.81 | 0.67–0.89 |
| Shame - Victim intentional | 2.52 | 0.88 | 2.69 | 0.92 | 6.75 | 0.78 | 0.60–0.87 |
| Shame - Victim unintentional | 1.57 | 0.61 | 1.62 | 0.62 | 3.18 | 0.79 | 0.63–0.88 |
| Bad - Agent intentional | 1.84 | 0.63 | 1.89 | 0.56 | 2.72 | 0.82 | 0.67–0.90 |
| Bad - Agent unintentional | 2.28 | 0.71 | 2.29 | 0.63 | 0.44 | 0.85 | 0.74–0.92 |
| Bad - Victim intentional | 2.48 | 0.55 | 2.41 | 0.61 | -2.82 | 0.80 | 0.65–0.89 |
| Bad - Victim unintentional | 3.06 | 0.60 | 3.00 | 0.59 | -1.96 | 0.83 | 0.71–0.91 |

**Legend:** Mean and SD are reported for baseline and retest sessions (3-5 weeks) along with Intraclass Correlation Coefficient (ICC) and their 95% Confidence Interval (95% CI). Test-retest bias is calculated as percentage change between first and second test: *Test-retest bias = ((retest-test)/test)*100.*

**Moral Emotions Task - Correlations**

| | *Age* | *Sex*[$] | *Education* | *IQ* | *Neuroticism*[a] | *TMD* |
|---|---|---|---|---|---|---|
| Guilt - Agent intentional | 0.21 | 0.08 | -0.09 | -0.08 | 0.06 | -0.07 |
| Guilt - Agent unintentional | 0.05 | 0.24 | 0.01 | -0.13 | 0.24 | 0.10 |
| Guilt - Victim intentional | 0.05 | 0.20 | -0.15 | -0.01 | **0.28**** | 0.22 |
| Guilt - Victim unintentional | 0.09 | 0.05 | 0.09 | 0.01 | 0.12 | 0.07 |
| Shame - Agent intentional | 0.08 | 0.19 | -0.07 | -0.07 | 0.14 | -0.02 |
| Shame - Agent unintentional | -0.04 | **0.29**** | 0.02 | -0.20 | **0.22**** | 0.17 |
| Shame - Victim intentional | -0.07 | 0.19 | -0.07 | -0.18 | **0.29**** | 0.18 |
| Shame - Victim unintentional | 0.01 | 0.06 | 0.10 | -0.03 | **0.28**** | 0.22 |
| Bad - Agent intentional | -0.21 | -0.13 | 0.05 | 0.04 | 0.01 | 0.10 |
| Bad - Agent unintentional | 0.01 | -0.23 | 0.08 | 0.18 | -0.08 | 0.07 |
| Bad - Victim intentional | 0.02 | -0.09 | 0.12 | 0.04 | -0.13 | -0.06 |
| Bad - Victim unintentional | 0.05 | -0.02 | 0.19 | 0.04 | -0.26 | -0.09 |

**Legend:** Correlations between Moral Emotions Task (ME) outcomes and age, sex, education indexed with the Family History Assessment Module (OS-FHAM) on a five-point likert scale, IQ score assessed with the Reynolds Intellectual Screening Test (RIST), total mood disturbance (TMD) indexed with the Profile of Mood Scale (POMS), and trait Neuroticism indexed with the NEO Personality Inventory-Revised (NEO PI-R, n = 93) and the NEO Personality Inventory 3 (NEO PI-3, n = 6). Correlation coefficients are reported as Spearman's rho; only $p$-values < .01 are considered significant and are marked in bold. ** = $p$ < .01. [$] A negative rho value indicates males score higher while a positive rho value indicates females score higher. [a] N = 99 due to missing data from one participant.

# 9. Social Information Preference Task

### Task description

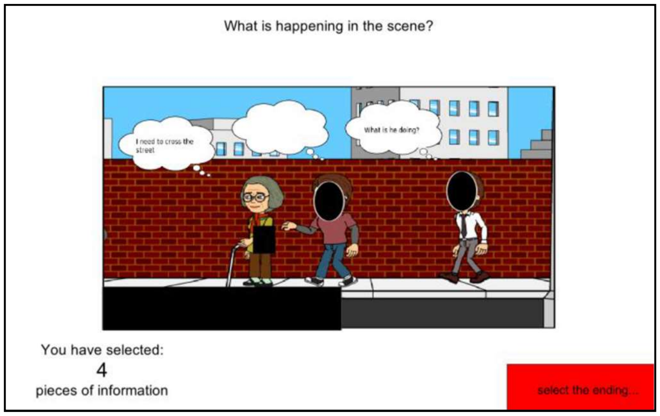


The Social Information Preference Task (SIP) assesses information sampling for interpretation of social situations. A series of social scenarios are shown in which nine pieces of information are hidden; three thought bubbles, three faces, and three object/facts. The participant is allowed to choose four pieces of information to help them decide what is happening in the situation. They are then asked to choose between three different interpretations (a positive, a neutral, and a negative) as well as rate how confident they are in their choice on a scale from 1 (‘not at all’) to 7 (‘very much so’). All outcomes are equally plausible and there are no right or wrong answers. The task consists of 18 scenarios.

**Main outcomes** (reported in the main article)

- *Type of information chosen* – The percentage of thoughts, faces, and facts chosen

### Secondary outcomes

- *Type of outcomes chosen* – The percentage of positive, neutral, and negative outcomes chosen
- *Confidence in choice of outcome* – Average confidence rating for positive, neutral, and negative outcome choices

**Social Information Preference Task – Descriptive data**

| | *Mean* | *SD* | *Median* | *IQR* | *Range* | *Skewness* | *Floor effect* | *Ceiling effect* |
|---|---|---|---|---|---|---|---|---|
| Outcome (%) - Positive | *38.44* | *12.09* | 40.62 | 12.50 | 6.25–75.00 | -0.14 | 0% | 0% |
| Outcome (%) - Neutral | *33.56* | *10.90* | 31.25 | 14.06 | 12.50–62.50 | 0.44 | 0% | 0% |
| Outcome (%) - Negative | *28.00* | *12.67* | 28.12 | 18.75 | 0.00–56.25 | 0.32 | 1% | 0% |
| Confidence in outcome (%) - Positive | 4.90 | 0.83 | 4.88 | 1.16 | 2.38–6.50 | -0.48 | 0% | 0% |
| Confidence in outcome (%) - Neutral | 4.74 | 0.93 | 4.80 | 1.18 | 2.86–7.00 | 0.04 | 0% | 1% |
| Confidence in outcome (%) - Negative | 4.85 | 1.03 | 4.83 | 1.23 | 2.00–7.00 | -0.26 | 0% | 3% |

**Legend:** Mean, SD, median, interquartile range, range, and skewness are shown. Note, for outcomes with non-parametric data distribution, mean and SD are denoted in cursive gray indicating the median and interquartile range should be used as reference. Floor and ceiling effects are shown as percentage of test subjects who achieved minimum (floor) or maximum (ceiling) scores.

**Social Information Preference Task – Test-retest reliability**

| | **Baseline (n = 49)** | | **Retest (n = 49)** | | *Test-retest bias (%)* | *ICC* | *95% CI* |
|---|---|---|---|---|---|---|---|
| | *Mean* | *SD* | *Mean* | *SD* | | | |
| Outcome (%) - Positive | 37.24 | 13.26 | 40.31 | 14.66 | 8.24 | 0.65 | 0.38–0.80 |
| Outcome (%) - Neutral | 35.84 | 11.47 | 37.50 | 11.76 | 4.63 | 0.33 | -0.20–0.62 |
| Outcome (%) - Negative | 26.91 | 12.51 | 22.19 | 13.32 | -17.54 | 0.77 | 0.55–0.87 |
| Confidence in outcome (%) - Positive | 4.85 | 0.86 | 4.86 | 0.84 | 0.21 | 0.58 | 0.26–0.77 |
| Confidence in outcome (%) - Neutral | 4.59 | 0.92 | 4.75 | 0.83 | 3.49 | 0.58 | 0.27–0.76 |
| Confidence in outcome (%) - Negative | 4.80 | 1.10 | 4.68 | 1.11 | -2.50 | 0.34 | -0.18–0.63 |

**Legend:** Mean and SD are reported for baseline and retest sessions (3-5 weeks) along with Intraclass Correlation Coefficient (ICC) and their 95% Confidence Interval (95% CI). Test-retest bias is calculated as percentage change between first and second test: *Test-retest bias = ((retest-test)/test)*100.*

**Social Information Preference Task – Correlations**

| | *Age* | *Sex*[$] | *Education* | *IQ* | *Neuroticism*[a] | *TMD* |
|---|---|---|---|---|---|---|
| Outcome (%) - Positive | 0.19 | -0.16 | -0.06 | -0.11 | 0.01 | -0.08 |
| Outcome (%) - Neutral | **0.29**** | -0.07 | -0.04 | 0.24 | -0.11 | -0.08 |
| Outcome (%) - Negative | **-0.42***** | 0.23 | 0.07 | -0.08 | 0.12 | 0.17 |
| Confidence in outcome (%) - Positive | 0.06 | -0.06 | -0.07 | -0.02 | -0.10 | -0.01 |
| Confidence in outcome (%) - Neutral | 0.05 | -0.10 | 0.04 | -0.03 | -0.14 | -0.10 |
| Confidence in outcome (%) - Negative | 0.01 | -0.06 | 0.06 | 0.09 | -0.16 | -0.21 |

**Legend:** Correlations between Social Information Preference task (SIP) outcomes and age, sex, education indexed with the Family History Assessment Module (OS-FHAM) on a five-point likert scale, IQ score assessed with the Reynolds Intellectual Screening Test (RIST), total mood disturbance (TMD) indexed with the Profile of Mood Scale (POMS), and trait Neuroticism indexed with the NEO Personality Inventory-Revised (NEO PI-R, n = 93) and the NEO Personality Inventory 3 (NEO PI-3, n = 6). Correlation coefficients are reported as Spearman's rho; only *p*-values <.01 are considered significant and are marked in bold. ** = $p < .01$, *** = $p < .001$. [$] A negative rho value indicates males score higher while a positive rho value indicates females score higher. [a] N = 99 due to missing data from one participant.

# 10. Prisoner's Dilemma

## Task description

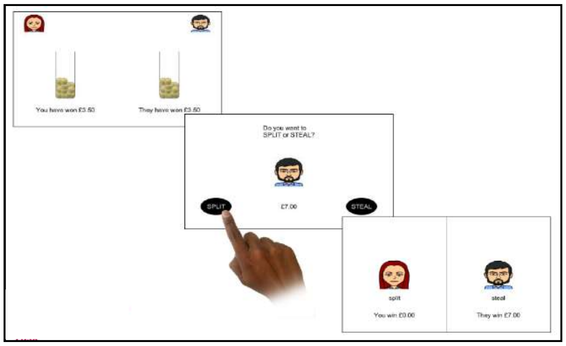


The Prisoner's Dilemma task (PD) assesses cooperative behavior. At the beginning of each trial, the participant and a computer opponent complete a small task to collect money which is then pooled; a third of the time, the participant contributes more, a third of the time both parties contribute equally, and a third of the time the opponent contributes more. The participant is asked whether they wish to split or steal the pooled winnings. If both the participant and the opponent decide to split, they each get half, if they both decide to steal, neither of them get anything. If one player decides to steal and the other to split, the player who stole gets the whole sum. The participant is faced with three different opponents exhibiting different strategies: Cooperative (always splits), tit-for-two-tat (starts with a split and the changes behavior if the participant steals two times consecutively), and aggressive (tit-for-tat, starting with a steal). There are nine trials for each opponent type, equaling 27 trials in total.

**Main outcomes** (reported in the main article)

- *Proportion of steals* – Proportion of trials (%) in which the participant chooses to steal for each type of opponent (cooperative, tit-for-two-tat, aggressive)

## Secondary outcomes

- *Proportion of steals based on contribution* – Proportion of trials (%) in which the participant chooses to steal from each type of opponent (cooperative, tit-for-tat, aggressive) for each level of contribution (more, equal, less)

**Prisoner's Dilemma – Descriptive data**

| | *Mean* | *SD* | *Median* | *IQR* | *Range* | *Skewness* | *Floor effect* | *Ceiling effect* |
|---|---|---|---|---|---|---|---|---|
| Steals cooperative (%) - Player contributes more | *23.33* | *34.98* | 0.00 | 33.33 | 0–100 | 1.22 | 63% | 11% |
| Steals cooperative (%) - Equal contribution | *13.33* | *28.43* | 0.00 | 0.00 | 0–100 | 2.11 | 78% | 6% |
| Steals cooperative (%) - Player contributes less | *25.00* | *37.12* | 0.00 | 33.33 | 0–100 | 1.14 | 63% | 14% |
| Steals tit-for-two-tat (%) - Player contributes more | *28.00* | *38.99* | 0.00 | 66.67 | 0–100 | 0.94 | 61% | 16% |
| Steals tit-for-two-tat (%) - Equal contribution | *21.00* | *34.38* | 0.00 | 33.33 | 0–100 | 1.36 | 68% | 10% |
| Steals tit-for-two-tat (%) - Player contributes less | *27.67* | *37.32* | 0.00 | 66.67 | 0–100 | 0.92 | 59% | 13% |
| Steals aggressive (%) - Player contributes more | *38.33* | *37.72* | 33.33 | 66.67 | 0–100 | 0.38 | 41% | 16% |
| Steals aggressive (%) - Equal contribution | *30.33* | *35.17* | 33.33 | 66.67 | 0–100 | 0.76 | 49% | 11% |
| Steals aggressive (%) - Player contributes less | *36.33* | *39.10* | 33.33 | 66.67 | 0–100 | 0.44 | 48% | 16% |

**Legend:** Mean, SD, median, interquartile range, range, and skewness are shown. Note, for outcomes with non-parametric data distribution, mean and SD are denoted in cursive gray indicating the median and interquartile range should be used as reference. Floor and ceiling effects are shown as percentage of test subjects who achieved minimum (floor) or maximum (ceiling) scores. Note, floor effects are defined as players who never steals from the opponent while ceiling effects are defined as players who always steals from opponent.

**Prisoner's Dilemma – Test-retest reliability**

| | Baseline (n = 49) | | Retest (n = 49) | | *Test-retest bias (%)* | *ICC* | *95% CI* |
|---|---|---|---|---|---|---|---|
| | *Mean* | *SD* | *Mean* | *SD* | | | |
| Steals cooperative (%) - Player contributes more | 17.69 | 28.95 | 21.77 | 33.02 | 23.06 | 0.50 | 0.12–0.72 |
| Steals cooperative (%) - Equal contribution | 8.84 | 24.32 | 13.61 | 27.99 | 53.96 | 0.37 | -0.12–0.64 |
| Steals cooperative (%) - Player contributes less | 17.69 | 31.26 | 17.69 | 32.70 | 0.00 | 0.71 | 0.49–0.84 |
| Steals tit-for-two-tat (%) - Player contributes more | 23.13 | 36.77 | 19.05 | 29.66 | -17.64 | 0.66 | 0.40–0.81 |
| Steals tit-for-two-tat (%) - Equal contribution | 13.61 | 27.99 | 14.29 | 28.05 | 5.00 | 0.63 | 0.34–0.79 |
| Steals tit-for-two-tat (%) - Player contributes less | 19.73 | 30.37 | 16.33 | 28.16 | -17.23 | 0.39 | -0.09–0.66 |
| Steals aggressive (%) - Player contributes more | 31.97 | 38.47 | 29.25 | 32.37 | -8.51 | 0.63 | 0.34–0.79 |
| Steals aggressive (%) - Equal contribution | 25.85 | 32.11 | 21.09 | 31.69 | -18.41 | 0.58 | 0.26–0.76 |
| Steals aggressive (%) - Player contributes less | 30.61 | 38.99 | 34.69 | 39.06 | 13.33 | 0.54 | 0.18–0.74 |

**Legend:** Mean and SD are reported for baseline and retest sessions (3-5 weeks) along with Intraclass Correlation Coefficient (ICC) and their 95% Confidence Interval (95% CI). Test-retest bias is calculated as percentage change between first and second test: *Test-retest bias = ((retest-test)/test)*100.*

**Prisoner's Dilemma - Correlations**

| | *Age* | *Sex*[$] | *Education* | *IQ* | *Neuroticism*[a] | *TMD* | *Motivation* | *Diligence* |
|---|---|---|---|---|---|---|---|---|
| Steals cooperative (%) - Player contributes more | -0.08 | -0.17 | -0.08 | -0.02 | 0.08 | -0.05 | -0.10 | 0.01 |
| Steals cooperative (%) - Equal contribution | -0.20 | -0.20 | 0.17 | 0.03 | -0.03 | -0.09 | 0.03 | 0.01 |
| Steals cooperative (%) - Player contributes less | -0.15 | -0.08 | -0.08 | -0.13 | 0.04 | -0.11 | 0.00 | 0.06 |
| Steals tit-for-two-tat (%) - Player contributes more | -0.06 | -0.25 | -0.09 | -0.08 | 0.11 | -0.01 | 0.01 | 0.09 |
| Steals tit-for-two-tat (%) - Equal contribution | -0.13 | -0.24 | 0.03 | -0.07 | 0.08 | 0.02 | -0.03 | 0.02 |
| Steals tit-for-two-tat (%) - Player contributes less | -0.04 | -0.20 | 0.00 | -0.15 | 0.05 | -0.04 | 0.06 | 0.12 |
| Steals aggressive (%) - Player contributes more | -0.05 | -0.18 | 0.07 | -0.06 | 0.06 | -0.03 | 0.06 | 0.12 |
| Steals aggressive (%) - Equal contribution | -0.01 | **-0.32**** | 0.03 | 0.08 | 0.07 | -0.02 | 0.08 | 0.08 |
| Steals aggressive (%) - Player contributes less | -0.07 | -0.20 | 0.01 | -0.02 | 0.10 | 0.01 | 0.12 | 0.13 |

**Legend:** Correlations between Prisoner's Dilemma task outcomes and age, sex, education indexed with the Family History Assessment Module (OS-FHAM) on a five-point likert scale, IQ score assessed with the Reynolds Intellectual Screening Test (RIST), total mood disturbance (TMD) indexed with the Profile of Mood Scale (POMS), and trait Neuroticism indexed with the NEO Personality Inventory-Revised (NEO PI-R, n = 93) and the NEO Personality Inventory 3 (NEO PI-3, n = 6). Correlations between self-reported motivation and diligence are also shown. Correlation coefficients are reported as Spearman's rho; only $p$-values < 0.01 are considered significant and are marked in bold. ** = $p < .01$. [$] A negative rho value indicates males score higher while a positive rho value indicates females score higher. [a] N = 99 due to missing data from one participant.

# 11. Ultimatum Game

## Task description

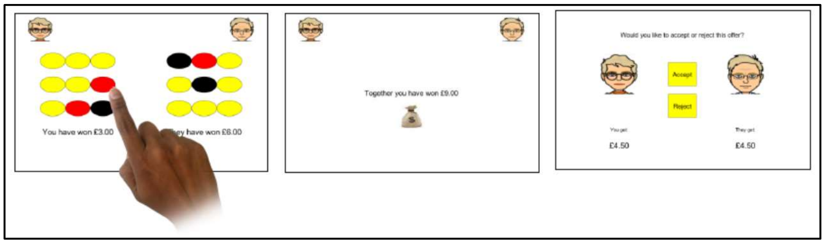


The Ultimatum Game (UG) assesses sensitivity to fairness. At the beginning of each trial, the participant and a computer opponent complete a small task to collect money which is then pooled; a third of the time, the participant contributes more, a third of the time both parties contribute equally, and a third of the time the opponent contributes more. Some of the time, the participant decides how the money is split and other times the opponent decides how the money is split. The opponent's offer ranges in fairness (50% offer, 40% offer, 35% offer, 30% offer, 25% offer, 20% offer, 10% offer) and the participant can decide to accept the offer and get the proposed amount or reject the offer and get no money. The participant decides the split in 15 trials and the opponent decides the split in 36 trials, equaling a total of 51 trials.

**Main outcomes** (reported in the main article)

- *Proportion of accepted offers* – Proportion of trials (%) in which the participant accepts the proposed offer

**Secondary outcomes**

- *Proportion of accepted offers at different fairness levels* – Proportion of offers (%) accepted at different fairness levels (50%, 40%, 35%, 30%, 25%, 20%, 10%).
- *Average offer proposed* – The average offer proposed by the participant

**Ultimatum Game – Descriptive data**

| | *Mean* | *SD* | *Median* | *IQR* | *Range* | *Skewness* | *Floor effect* | *Ceiling effect* |
|---|---|---|---|---|---|---|---|---|
| Acceptance rate (%) - 50% offer | *98.33* | *7.30* | 100.00 | 0.00 | 66.67–100 | -4.19 | 0% | 95% |
| Acceptance rate (%) - 40% offer | *79.83* | *29.23* | 100.00 | 33.33 | 0–100 | -1.30 | 3% | 57% |
| Acceptance rate (%) - 35% offer | *74.00* | *33.28* | 91.67 | 50.00 | 0–100 | -0.99 | 7% | 50% |
| Acceptance rate (%) - 30% offer | *61.00* | *35.31* | 66.67 | 66.67 | 0–100 | -0.35 | 11% | 31% |
| Acceptance rate (%) - 25% offer | *49.00* | *37.44* | 41.67 | 66.67 | 0–100 | 0.11 | 19% | 22% |
| Acceptance rate (%) - 20% offer | *39.67* | *39.91* | 33.33 | 87.50 | 0–100 | 0.57 | 32% | 25% |
| Acceptance rate (%) - 10% offer | *25.67* | *39.03* | 0.00 | 33.33 | 0–100 | 1.16 | 64% | 18% |
| Average offer proposed (%) | *37.93* | *9.42* | 39.67 | 14.50 | 20–50 | -0.47 | 4% | 12% |

**Legend:** Mean, SD, median, interquartile range, range, and skewness are shown. Note, for outcomes with non-parametric data distribution, mean and SD are denoted in cursive gray indicating the median and interquartile range should be used as reference. Floor and ceiling effects are shown as percentage of test subjects who achieved minimum (floor) or maximum (ceiling) scores. Note, for acceptance rate outcomes floor effect describes players who rejected all offers (0%) while ceiling effect describes players who accepted all offers (100%). For the average offer proposed outcome, floor effect describes players who made only the lowest offers (20%) while ceiling effects describes players who made only the highest offer (50%).

**Ultimatum Game – Test-retest reliability**

| | Baseline (n = 49) | | Retest (n = 49) | | *Test-retest bias (%)* | *ICC* | *95% CI* |
|---|---|---|---|---|---|---|---|
| | *Mean* | *SD* | *Mean* | *SD* | | | |
| Acceptance rate (%) - 50% offer | 98.64 | 6.66 | 98.64 | 6.66 | 0.00 | 0.65 | 0.38–0.80 |
| Acceptance rate (%) - 40% offer | 77.55 | 30.72 | 85.03 | 29.71 | 9.65 | 0.81 | 0.66–0.89 |
| Acceptance rate (%) - 35% offer | 74.15 | 34.70 | 81.63 | 31.41 | 10.09 | 0.86 | 0.74–0.92 |
| Acceptance rate (%) - 30% offer | 60.20 | 34.33 | 74.83 | 34.55 | 24.30 | 0.75 | 0.50–0.87 |
| Acceptance rate (%) - 25% offer | 47.62 | 35.19 | 67.69 | 36.07 | 42.15 | 0.68 | 0.32–0.84 |
| Acceptance rate (%) - 20% offer | 35.37 | 36.90 | 60.54 | 41.48 | 71.16 | 0.59 | 0.18–0.79 |
| Acceptance rate (%) - 10% offer | 20.41 | 34.57 | 35.37 | 43.78 | 73.30 | 0.64 | 0.35–0.79 |
| Average offer proposed (%) | 38.78 | 8.70 | 32.84 | 11.39 | -15.32 | 0.72 | 0.32–0.87 |

**Legend:** Mean and SD are reported for baseline and retest sessions (3-5 weeks) along with Intraclass Correlation Coefficient (ICC) and their 95% Confidence Interval (95% CI). Test-retest bias is calculated as percentage change between first and second test: *Test-retest bias = ((retest-test)/test)*100.*

**Ultimatum Game - Correlations**

| | *Age* | *Sex*[$] | *Education* | *IQ* | *Neuroticism*[a] | *TMD* | *Motivation* | *Diligence* |
|---|---|---|---|---|---|---|---|---|
| Acceptance rate (%) - 50% offer | -0.15 | -0.05 | -0.13 | -0.12 | 0.06 | 0.04 | 0.25 | 0.03 |
| Acceptance rate (%) - 40% offer | -0.20 | 0.03 | 0.19 | -0.09 | 0.08 | 0.08 | -0.02 | -0.18 |
| Acceptance rate (%) - 35% offer | -0.18 | 0.08 | 0.22 | -0.11 | 0.14 | 0.16 | 0.08 | -0.05 |
| Acceptance rate (%) - 30% offer | -0.12 | 0.08 | 0.12 | -0.02 | 0.07 | 0.13 | -0.02 | -0.19 |
| Acceptance rate (%) - 25% offer | -0.15 | 0.04 | 0.16 | -0.01 | 0.03 | 0.19 | -0.04 | -0.24 |
| Acceptance rate (%) - 20% offer | -0.12 | 0.16 | 0.08 | -0.10 | 0.01 | 0.19 | -0.03 | -0.21 |
| Acceptance rate (%) - 10% offer | -0.12 | 0.04 | 0.08 | -0.09 | 0.01 | 0.13 | -0.10 | **-0.27**** |
| Average offer proposed (%) | 0.23 | 0.04 | -0.04 | 0.20 | -0.11 | -0.06 | 0.00 | 0.18 |

**Legend:** Correlations between Prisoner's Dilemma task outcomes and age, sex, education indexed with the Family History Assessment Module (OS-FHAM) on a five-point likert scale, IQ score assessed with the Reynolds Intellectual Screening Test (RIST), total mood disturbance (TMD) indexed with the Profile of Mood Scale (POMS), and trait Neuroticism indexed with the NEO Personality Inventory-Revised (NEO PI-R, n = 93) and the NEO Personality Inventory 3 (NEO PI-3, n = 6). Correlations between self-reported motivation and diligence are also shown. Correlation coefficients are reported as Spearman's rho; only *p*-values < .01 are considered significant and are marked in bold. ** = $p < .01$. [$] A negative rho value indicates males score higher while a positive rho value indicates females score higher. [a] N = 99 due to missing data from one participant.

## Exploratory Factor Analysis

Table A shows the factors and item loadings of an exploratory factor analysis. The analysis was conducted using principal axis factoring with Varimax rotation; only items with loadings > 0.3 are reported. Measures of sampling adequacy were acceptable: the Kaiser-Meyer-Olkin value was sufficient (KMO = 0.53) and Bartlett's test of sphericity was significant (*p* < .001) indicating that the data was suitable for structure detection. Using an eigen-value cutoff of 1, the factor analysis suggested 13 factors (see Figure A for scree plot).

**Table A. Factor loadings for EMOTICOM tasks on factors 1-13**

| | *1* | *2* | *3* | *4* | *5* | *6* | *7* | *8* | *9* | *10* | *11* | *12* | *13* |
|---|---|---|---|---|---|---|---|---|---|---|---|---|---|
| **Emotional Intensity Morphing Task: Increase condition** | | | | | | | | | | | | | |
| Detection threshold - Happy | 0.62 | | | | | 0.57 | | | | | | | |
| Detection threshold - Sad | 0.76 | | | | | | | | | | | | |
| Detection threshold - Angry | 0.79 | | | | | | | | | | | | |
| Detection threshold - Fearful | 0.83 | | | | | | | | | | | | |
| Detection threshold - Disgusted | 0.85 | | | | | | | | | | | | |
| **Emotional Intensity Morphing Task: Decrease condition** | | | | | | | | | | | | | |
| Detection threshold - Happy | | 0.42 | | | | | | -0.57 | | | | | -0.31 |
| Detection threshold - Sad | | 0.64 | | | | | | | | | | | |
| Detection threshold - Angry | | 0.68 | | | | | | | | | | | |
| Detection threshold - Fearful | | 0.71 | | | | | | | | | | | |
| Detection threshold - Disgusted | | 0.80 | | | | | | | | | | | |
| **Prisoner's Dilemma** | | | | | | | | | | | | | |
| Proportion steals (%) - Cooperative | | | 0.77 | | | | | | | | | | |
| Proportion steals (%) - Tit-for-two-tat | | | 0.84 | | | | | | | | | | |
| Proportion steals (%) - Aggressive | | | 0.86 | | | | | | | | | | |
| **Affective Go/NoGo** | | | | | | | | | | | | | |

| | | | | | | | | | | | | | |
|---|---|---|---|---|---|---|---|---|---|---|---|---|---|
| d-prime - Happy/Neutral | | | | 0.33 | | | | | | | | | |
| d-prime - Happy/Sad | | | | 0.49 | | | | | | | | | |
| d-prime - Neutral/Happy | | | | 0.57 | | | | | | | | | |
| d-prime - Neutral/Sad | | | | 0.63 | | | | | | | | | 0.31 |
| d-prime - Sad/Happy | | | | 0.64 | | | | | | | | | |
| d-prime - Sad/Neutral | | | | 0.39 | | | | | | | | | |
| **Moral Emotions Task** | | | | | | | | | | | | | |
| Guilt - Agent | | | | | 0.88 | | | | | | | | |
| Guilt - Victim | | | | | | | | | 0.80 | | | | |
| Shame - Agent | | | | | 0.84 | | | | | | | | |
| Shame - Victim | | | | | | | | | 0.83 | | | | |
| **Emotional Face Recognition Task: Face version** | | | | | | | | 0.61 | | | | | |
| Accuracy (%) - Happy | | | | | | | | | | | | | |
| Accuracy (%) - Sad | | | | | | 0.31 | | | | 0.41 | | | |
| Accuracy (%) - Angry | | | | | | | | | | 0.72 | | | |
| Accuracy (%) - Fearful | | | | | | 0.40 | | | | 0.51 | | | |
| **Emotional Face Recognition Task: Eyes version** | | | | | | | | | | | | | |
| Accuracy (%) - Happy | | | | | | | | 0.73 | | | | | |
| Accuracy (%) - Sad | | | | | | 0.70 | | | | | | | |
| Accuracy (%) - Angry | | | | | | | | | | 0.40 | | | |
| Accuracy (%) - Fearful | | | | | | 0.57 | | | | 0.33 | | | |
| **Social Information Preference Task** | | | | | | | | | | | | | |
| Information (%) - Thoughts | | | | | | | -0.88 | | | | | | |
| Information (%) - Faces | | | | | | | 0.77 | | | | | | |
| Information (%) – Facts* | | | | | | | | | | | | | |
| **Reinforcement Learning Task**[a] | | | | | | | | | | | | | |
| Alpha - Win condition | | | | | | | | | | | | | |
| Alpha - Loss condition | | | | | | | | | | | | | |
| **Monetary Incentive Reward Task** | | | | | | | | | | | | | |
| Reaction time (ms) - Win | | | | | | | | | | | -0.32 | | 0.34 |

| | | | | | | | | | | | | | |
|---|---|---|---|---|---|---|---|---|---|---|---|---|---|
| Reaction time (ms) - Loss | | | | | | | | | | | | 0.73 | |
| **Progressive Ratio Task** | | | | | | | | | | | | | |
| Breakpoint (trials) | | | | | | | | | | | | | |
| **Adapted Cambridge Gambling Task** | | | | | | | | | | | | | |
| Risk adjustment - Win condition | | | | | | | | | | | | | |
| Risk adjustment - Loss condition | | | | | | | | | | | 0.83 | | |
| **Ultimatum Game** | | | | | | | | | | | | | |
| Average acceptance rate (%) | | | | | | | | | | | 0.36 | | 0.30 |

* We removed one of the three outcomes (‘Facts’) from the *Social Information Preference task* from the analysis as it is redundant; the sum of the three outcomes always equals 100%, meaning the value of one can always be inferred from the other two.

**Figure A. Scree plot**

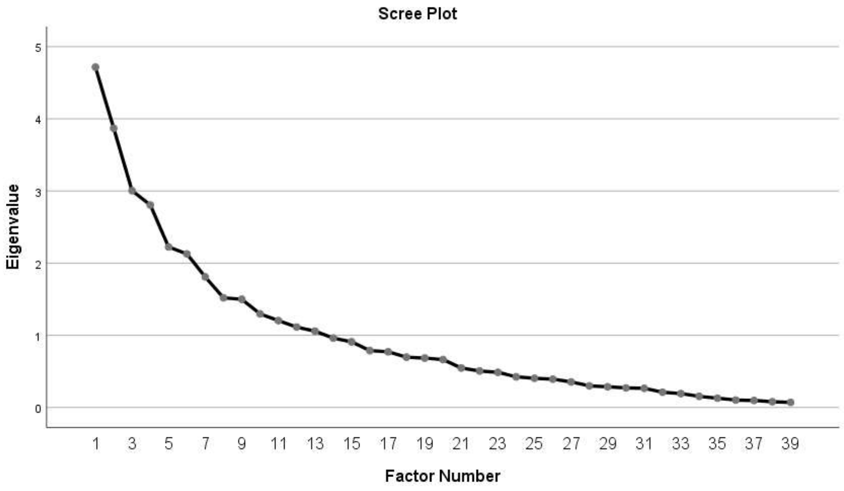

Supplement: Supplementary file 1 [file Data_Sheet_1.PDF]
